# Functional connectivity and characteristics of cortical brain networks of elderly individuals under different motor cognitive tasks based on functional near-infrared spectroscopy

**DOI:** 10.3389/fnhum.2025.1563338

**Published:** 2025-04-24

**Authors:** Shanshan Jiang, Zhiqing Qiu, Xiaoqing Cai, Tingting You, Xinyu Fu, Guanzhou Chen, Haoda Li, Haining Ou

**Affiliations:** ^1^The Fifth Affiliated Hospital of Guangzhou Medical University, Guangzhou, China; ^2^The Fifth Clinical College, Guangzhou Medical University, Guangzhou, China; ^3^Maoming People's Hospital, Maoming, China; ^4^The First Affiliated Hospital of Hunan University of Medicine, Huaihua, China; ^5^The Second Affiliated Hospital of Guangzhou University of Chinese Medicine, Guangzhou, China

**Keywords:** functional near infrared spectroscopy, healthy brain ageing, motor cognitive dual task, functional connectivity, cortical brain networks

## Abstract

**Objective:**

This study aimed to investigate age-related changes in brain functional connectivity during various motor and cognitive tasks, providing evidence for evaluating and intervening in brain aging.

**Methods:**

15 elderly participants (ELD) and 30 young controls (YOU) were assessed. fNIRS haemodynamic responses were recorded during the Purdue nail board motor task, continuous minus 7 cognitive task, and motor-cognitive dual task. Differences in brain activation, functional connectivity, integral values, and barycentre values between the groups were compared using oxygenated haemoglobin (HbO) concentrations over time.

**Results:**

The ELD group performed significantly worse than the YOU group (*p* < 0.05). ELD participants showed significantly lower activation in the LSMA during motor tasks (*p* < 0.05), the RDLPFC and LDLPFC during cognitive tasks (*p* < 0.05), and both RSMA and LSMA during dual tasks (*p* < 0.05). Functional connectivity between LDLPFC, RSMA, LSMA, and RDLPFC–LDLPFC, LSMA–RSMA in the ELD group was significantly lower than in the YOU group (*p* < 0.05). The ELD group also had lower connectivity in RSMA, RDLPFC–LDLPFC, and LSMA–RSMA during cognitive tasks (*p* < 0.05). The centre of gravity for the ELD group was significantly lower during dual tasks compared to the YOU group (*p* < 0.05). In cognitive tasks, the ELD group showed significantly lower RSMA centre of gravity and integral values compared to dual tasks (*p* = 0.05).

**Conclusion:**

Elderly individuals exhibit lower cortical brain connectivity than young people across various tasks. fNIRS-based cerebral haemodynamics provide a useful quantitative measure for evaluating age-related brain changes.

## 1 Introduction

Globally, the phenomenon of population ageing is gradually intensifying, and the ageing population will reach its peak in 2053, accounting for a quarter of the global population. Therefore, ageing-related motor and cognitive function degradation in elderly individuals have also become among the most important topics worldwide (Ferreira and Busatto, 2013). In the process of brain ageing, age-related changes in brain function are among the factors that cannot be ignored in overcoming the declines in cognitive and motor functions caused by brain ageing (Sperling et al., 2011).

Neurodegenerative processes during aging frequently manifest as diminished cognitive capacity, motor competency, or both. Critically, motor and cognitive systems are not functionally isolated. Ome studies have used walking speed, such as processing speed, memory ability and executive function (Paraskevoudi et al., 2018), to predict the cognitive ability of elderly individuals. This is due to the significant interaction between motor and cognitive functions at the neural circuit level, which is represented by the competitive resource allocation of executive function under the motor-cognitive dual task paradigm (Montero-Odasso et al., 2012; Yogev-Seligmann et al., 2008). A commonly used indicator to measure motor–cognitive dual tasks is dual-task cost (DT cost) (Yogev-Seligmann et al., 2008; Bayot et al., 2020). The motor–cognitive dual task induces the activation of the cognitive and motor networks at the same time, and both networks have common paths and specific paths, enabling us to explore the DT cost in the motor–cognitive dual task and the activation mode that changes with age.

In the past, research on the neural mechanism of the normal ageing process has relied mostly on functional magnetic resonance imaging (fMRI). Although this research tool is usually regarded as the gold standard for studying the neural mechanism of brain function, it has several limitations, such as requiring participants not to move (Lloyd-Fox et al., 2010). Functional near-infrared spectroscopy (fNIRS), a new noninvasive portable nerve detection technology, can reflect changes in oxygenated haemoglobin (HbO) and reduced haemoglobin (HbR) in the cerebral cortex in real time (Pinti et al., 2020). fNIRS overcomes the main defects of fMRI and can be applied to explore the haemodynamic changes in related brain activities during walking and other motor paradigms to further explore the neurological mechanism of brain ageing. Although fNIRS can be applied to many motor scenes, such as walking tasks, unlike fMRI, we found that in the actual operation process, there is still some signal interference and noise caused by walking itself, which may affect the experimental results. A more accurate assessment of brain ageing requires simple tasks with less noise (such as reducing the movement of the whole body). In this study, we creatively propose the use of fine upper limb movement task [Purdue pegboard task (PPT)] as a motor task, which has the advantage that can minimize head displacement (<2 cm translation) and greatly reduce the hemodynamic noise caused by movement. And the influence of fine motor performance has higher discriminant validity than gait parameters in distinguishing healthy aging (Vasylenko et al., 2018).

In addition, only limited research has focused on changes in brain activation characteristics when elderly individuals perform motor–cognitive dual tasks. Many studies have shown that the oxygenation level of the prefrontal cortex of elderly individuals increases from a single task to a motor–cognitive dual task, which indicates that motor–cognitive dual tasks require more prefrontal nerve resources than single tasks do (Doi et al., 2013; Holtzer et al., 2015). Previous studies on dual-task mainly focus on the frontal lobe as the brain area of interest, and few studies on other potential brain areas. However, recent studies have found that in addition to the prefrontal cortex, the auxiliary motor area and the premotor area cortex may also play an important role in dual-task walking in stroke patients. For example, Goh et al. investigated whether transcranial magnetic stimulation of the motor area could improve gait performance in young adults performing dual tasks (Goh et al., 2019). The results showed that when the excitability of the auxiliary motor area was enhanced, the subjects’ gait performance was better in completing the motor and cognitive dual task, suggesting that the auxiliary motor area played an important role in the dual task walking.

Although these functional studies provide important insights into the response of vascular dynamics to motor–cognitive dual tasks, further work is needed to evaluate the relationship between brain activation characteristics and motor–cognitive dual tasks. Most studies use the average signal intensity during the task, which is widely used to quantify the HbO change induced by the task, but some studies use the change rate or signal pattern as the characteristic quantity. For example, the characteristic quantity of psychiatric differentiation in emotional disorders is that the centre of gravity of the HbO signal waveform in the prefrontal area increases in the context of language fluency (Takizawa et al., 2014; Ehlis et al., 2014). Moreover, a domestic study noted that using the centre of gravity value as an objective evaluation index of the physical sensation of elderly people after stroke has certain application value (Jingya et al., 2022). These studies show that the application of fNIRS has adopted characteristic parameters rather than the average general signal strength. These characteristic evaluation parameters have not been applied to the study of brain ageing-related evaluation. The gravity centre value and integral value are quantitative indices reflecting the changes in the cerebral haemodynamic response with time. The integral value represents the total haemodynamic response during the task, which is an intensity index. The higher the value is, the greater the degree of neural activity related to cognitive tasks. The value of the centre of gravity represents the time required for the fNIRS signal to change to half during the whole experiment (including the rest state and the task state), and the smaller the value is, the faster and more relaxed the cortical response after the task is Takizawa et al. (2014), Ehlis et al. (2014), Jingya et al. (2022), and Wei et al. (2021). The high temporal resolution of fNIRS can not only detect dysfunction but also capture the time course of haemodynamic activation of each specific disease and help to differentiate diagnosis and evaluate intervention measures (Jingya et al., 2022).

In this study, fNIRS was used to monitor the dynamic changes in the brain network in young and old people during different motor and cognitive tasks and to explore the mechanism of age-related brain degeneration in the haemodynamics of the cognitive and motor areas (prefrontal lobe and supplementary motor area) of the cerebral cortex. We hypothesized that:(1) During the dual task, the individual pays more attention to the cognitive task than to the motor task, and thus the SMA activation is inhibited; (2) Under the dual task condition, the elderly group adopts local network compensation to maintain the basic task performance; (3) The advanced LDLPFC blood flow signal gravity value in the elderly with poor dual task performance suggests premature neurological resource exhaustion.

## 2 Materials and methods

This study was conducted in accordance with the principles stipulated in the Helsinki Declaration of the World Medical Association and was approved by the Ethics Committee of the Fifth Affiliated Hospital of Guangzhou Medical University (ethics number: GYWY-L2024-117). Moreover, the intervention program was registered at the International Clinical Trial Registration Center (registration number: ChiCTR2400092916). In addition, all the subjects signed informed consent forms before the experiment.

### 2.1 Research subjects

In this study, a nonrandomized controlled experimental design was adopted. From January 2024 to December 2024, 15 elderly people who met the inclusion criteria in the Department of Rehabilitation Medicine of the Fifth Affiliated Hospital of Guangzhou Medical University were selected as the elderly group (ELD group), and 30 young people were selected as the youth group (YOU group).

The inclusion criteria were as follows: (1) the youth group was 18–45 years old (including 18 and 45 years old), and the elderly group was 50–80 years old (including 50 and 80 years old); (2) the education level was > 6 years; (3) there were no obvious obstacles in vision and hearing; (4) the Mini-Mental State Examination (MMSE) score was ≥27; (5) the evaluation result of the Edinburgh Handedness Inventory was right-handed; and (6) the participants agreed to participate and signed the informed consent form.

The exclusion criteria were as follows: (1) serious neurological diseases or cerebral infarction in key parts; (2) severe mental illness; (3) severe failure of the heart, lung, liver, kidney or other important organ in the past; (4) uncontrollable hypertension, arrhythmia, severe coronary heart disease and poor control of diabetic complications; (5) wearing a pacemaker, having metal or other implants in the skull or having skull defects; (6) having other serious diseases that may affect the research; and (7) poor compliance, making it impossible to cooperate with the study.

The exit criterion was as follows: (1) during the trial, the patient experienced adverse reactions such as headache and nausea, which may have led to dangerous events. According to the doctor’s judgement, the clinical research could be stopped; that is, the clinical study of this case could be suspended.

### 2.2 Experimental methods

#### 2.2.1 Clinical evaluation

MMSE scale evaluation (Chua et al., 2019): MMSE, as the first choice for dementia screening, can comprehensively, accurately and quickly reflect the mental state and cognitive impairment of the subjects. It is sensitive to moderate and severe dementia and cognitive impairment in multiple cognitive domains. The subjects included in this study were all normal subjects with cognitive function, and their MMSE scores were ≥ 27.

Evaluation of the Edinburgh handedness scale (Milenkovic and Dragovic, 2013): The Edinburgh handedness scale is often used to evaluate handedness in neuroscience research. The subjects included in this study scored more than 40 and were judged to be right-handed.

#### 2.2.2 Experimental task design

The experimental scheme consists of three tasks: the partial Purdue nail board task (that is, the single motor task); the continuous minus 7 tasks (that is, the single cognitive task); and the partial Purdue nail board task plus the continuous minus 7 tasks (that is, motor–cognitive dual task).

Participants were asked to complete a single motor task, a single cognitive task and a motor-cognitive dual task. Participants drew a lottery to perform either the single motor task or the single cognitive task first, with a draw of 1 to perform the single motor task first and a draw of 2 to perform the single cognitive task first. The two single tasks were completed before the motor-cognitive dual task.

In addition, when the subjects sat quietly for 5 min, we also recorded the sitting resting state data of 10s as baseline.

##### 2.2.2.1 Single motor task

Partial Purdue nail board task: When they hear the system prompt “Please Start,” they perform part of the Purdue nail board task with their right hand; that is, they insert the nails in the plate into the corresponding holes in the top plate once with their right hand and then completely relax when the system prompts “Please Stop.” The assessor records the number of nails inserted in the partial Purdue nail board task as the evaluation index and restores the nails to the initial position. The partial Purdue nail board task and the complete relaxation time of both hands each lasted for 30 s and served as a block. The task consisted of three repeated blocks.

##### 2.2.2.2 Single cognitive task

Continuous minus 7 task: When prompted by the system “Please start,” the subjects start to calculate the continuous minus 7 from the randomly given two-digit number, verbally report the results after each minus 7, and then completely relax when prompted by the system “Please stop.” The appraisers record the correct/wrong numbers as the evaluation index. The continuous minus 7 task and the complete relaxation time each last for 30 s and serve as a block, and the task consists of three repeated blocks.

##### 2.2.2.3 Motor–cognitive dual task

Partial Purdue nail board + continuous minus 7 task: When the system prompts “Start,” the subjects perform the partial Purdue nail board task with their right hands, that is, they insert the nails in the plate into the corresponding holes in the top plate once with their right hands, and calculate the continuous minus 7 from the randomly given two-digit number, verbally report the results after each minus 7, and then completely relax when the system prompts “Please stop.” Before the official start, it was suggested that the two tasks should receive equal attention. The assessor records the number of nails inserted in the partial Purdue nail board task and the number of correct/wrong oral reports in the continuous minus 7 task as evaluation indicators and restores the nails to the initial position. The motor–cognitive dual task and the complete relaxation time each lasted for 30 s and served as a block. The task consisted of three repeated blocks.

The ratio of changes in cognitive and motor performance during dual task relative to single tasks was calculated to quantify the change in dual task performance (i.e., cognitive and motor costs, respectively). Cognitive and motor costs were calculated using the following formulars (Hall et al., 2011).


$$\begin{array}{l} \mathrm{Cognitvie}\;\mathrm{cost}\\ =\frac{\mathrm{Correct responses in minus}\;7-\mathrm{Correct responses in dual task}}{\mathrm{Correct responses in dual task}} \end{array}$$

$$\begin{array}{l} \mathrm{Motor}\;\mathrm{cost}\\ =\frac{\mathrm{Correct responses in Partial Purdue nail}-\mathrm{Correct responses in dual task}}{\mathrm{Correct responses in dual task}} \end{array}$$

Positive value indicates worse performance during dual task relative to single tasks (i.e., worse cognitive performance), whereas a negative value indicates better performance during dual task relative to single tasks (i.e., better cognitive performance). Cost sum was then calculated as the sum of cognitive and walking costs, reflecting the overall DT performance (Jayakody et al., 2020).

#### 2.2.3 fNIRS

**Figure 1:**
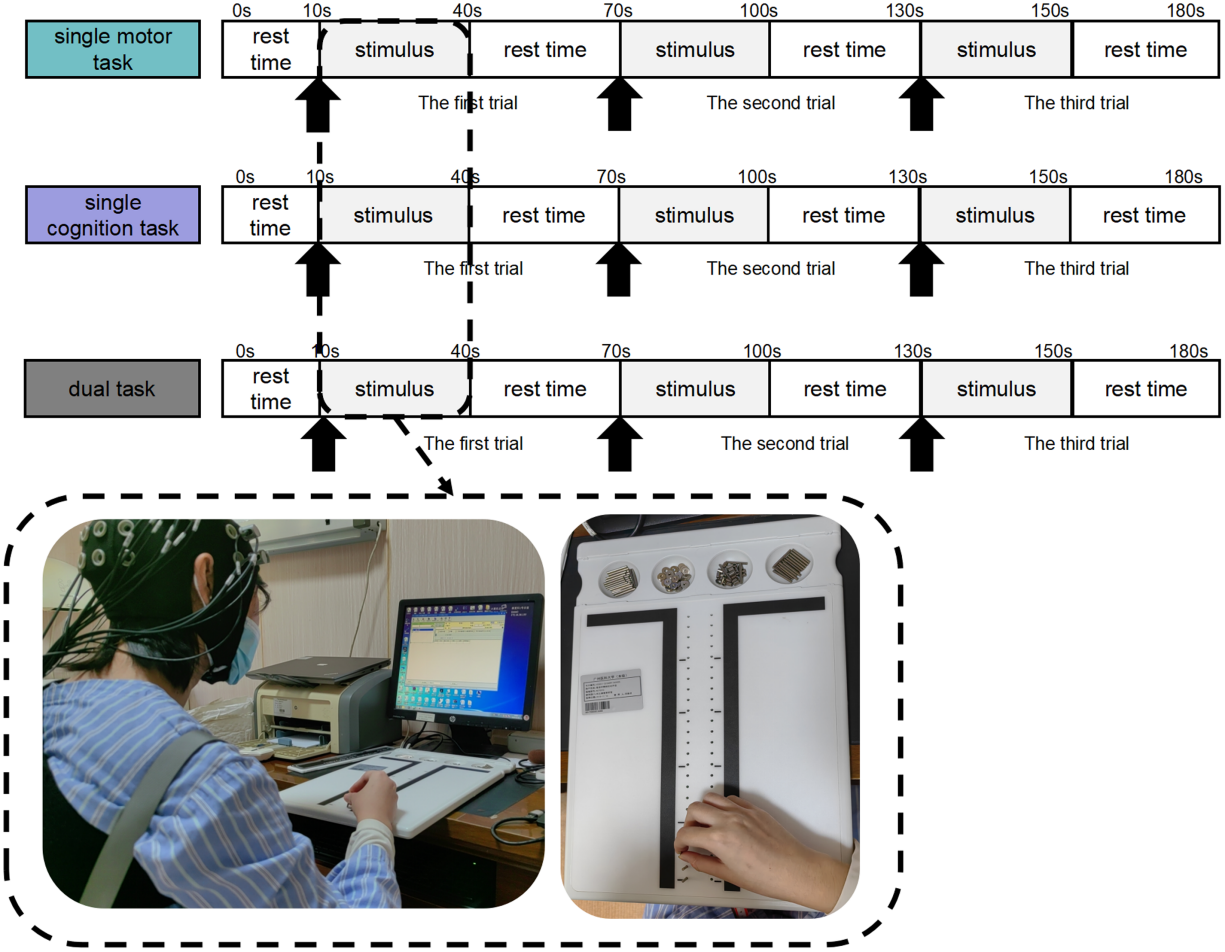
Motor–cognitive task setting process. Participants completed single motor task, single cognitive task, and motor-cognitive dual task in sequence. There was a rest period of 10s before the stimulation, as the baseline data. Each trial block included a stimulation period of 30 s and a rest period of 30 s, repeated 3 times in total.

In this study, synchronous fNIRS equipment was used to monitor haemodynamic changes during three tasks (Nirsmart, Danyang Huichuang Medical Equipment Co., Ltd., China). This equipment consists of 12 infrared light sources and 10 detectors, which constitute 24 channels, and their distribution positions are placed in the region of interest (ROI) according to the international 10–20 system (as shown in Figure 1). Each light source emits two kinds of infrared rays (wavelengths of 730 and 850 nm) to reach the cerebral cortex at approximately 5–8 mm, which are received by detectors with a spacing of 3 cm, and the haemodynamic response of the cerebral cortex is collected and recorded at a sampling rate of 10 Hz. The channel layout adopts a NirSpace (Danyang Huichuang Medical Equipment Co., Ltd., Danyang, China) positioning system and a three-dimensional digital brain area positioning instrument.

The calibration function of the instrument and corresponding template was used to ascertain the channels to fill exactly in correspondence with the 10/20 electrode positions. Each optode was attached to the surface of the skull using a custom-made hard plastic cap and covered with a black cloth to prevent penetration of ambient light. The hair was carefully swept away to ensure that the light tube touched the participant’s skin closely, thereby maximizing the efficiency of light coupling to the tissue.

**Figure 2:**
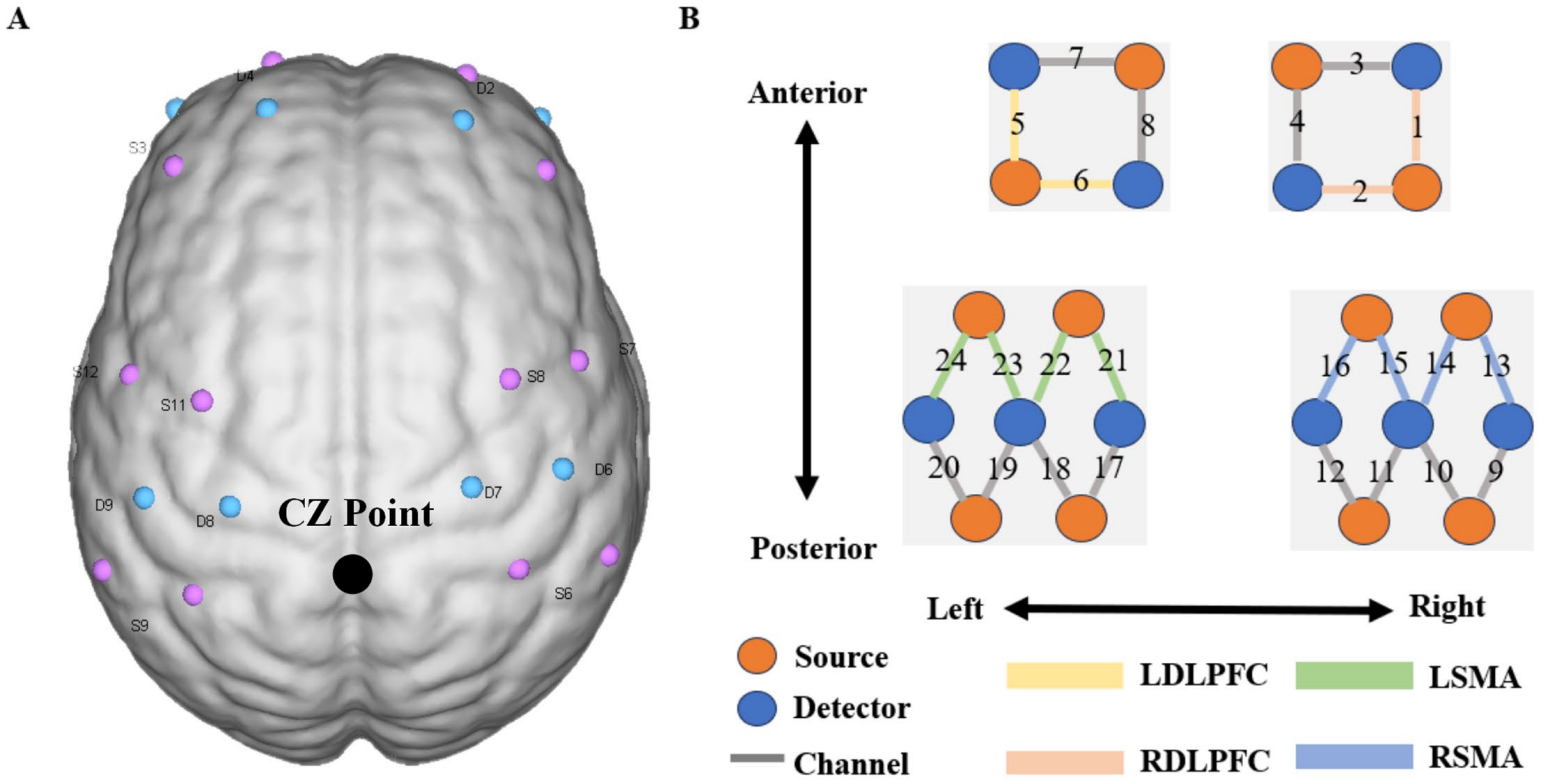
Near infrared spectrum test diagram. Figure A shows the layout design of fNIRS optoelectronic devices. The midline center point (Cz) is located at the midpoint of photodiode S9 and photodiode S6; Figure B shows the distribution diagram of 24 signal channels, where red represents the light source and blue. The detector is represented, the dark gray areas with numbers represent the channels, and the different colored areas represent brain function area.

The midline central point (Cz) was located at the midpoint of optode S9 and optode S6. The channel sets for regions of interest (ROI) were selected based on Brodmann areas (BA) and anatomical locations of cortical areas for each participant (Figure 2). The acquired coordinates were then transformed into the Montreal Neurological Institute (MNI) coordinates and further projected to the MNI standard brain template using a spatial registration approach in NirSpace (Danyang Huichuang Medical Equipment Co., Ltd., China). Based on Brodmann’s zoning, this study selected the left dorsolateral prefrontal cortex (LDLPFC), the right dorsolateral prefrontal cortex (RDLPFC), the left supplementary motor area (LSMA) and the right supplementary motor area (RSMA), for a total of 4 brain regions of interest. The LDLPFC consists of channels 5 and 6, the RDLPFC consists of channels 1 and 2, the LSMA consists of channels 21, 22, 23 and 24, and the RSMA consists of channels 13, 14, 15 and 16.

The selection of RDLPFC, LDLPFC, LSMA, and RSMA was based on their established roles in motor-cognitive integration and sensitivity to aging. The DLPFC is central to executive control and working memory, processes critical for dual-task performance and known to decline with age. The SMA is pivotal for motor planning and coordination, which are often impaired in aging populations, and it is integral to fine motor control due to its role in motor planning, sequencing, and bilateral coordination. SMA activation increases during precision-demanding tasks, such as the PPT, as it integrates sensory feedback and translates motor intentions into coordinated hand movements. Therefore, in this study, the left and right DLPFC and SMA were selected as ROI regions for exploration and analysis, in order to find the changes of brain function in these regions under single-task and dual-task states.

### 2.3 fNIRS data analysis

#### 2.3.1 fNIRS data preprocessing

In this experiment, NirSpark software (version 1.8.1) was used to preprocess fNIRS signals, which has been used in previous experiments (Chen et al., 2020). Inciuding four steps: 1. Elimination period: manually remove obvious motion artifacts, such as pulse type or cliff type jump (if there are no obvious artifacts, this step is skipped); 2. Motion artifact correction: remove motion artifacts based on the standard deviation of movement and spline interpolation. For example, if the difference between the maximum and minimum values in the time window of 0.5 s of light intensity is 6 times of the mean value, it is identified as motion artifacts and removed; 3. filtering: high-pass filtering is mainly used to filter out the low frequency drift unrelated to the experimental data, low-pass filtering is mainly used to filter out the high-frequency noise of the instrument, heartbeat or breathing introduced physiological noise; 4. blood oxygen conversion: The aim is to convert the obtained signal into a hemoglobin concentration change image. First of all, we eliminate this part of the time period and eliminate it by manual processing.

#### 2.3.2 Analysis of brain activation (Beta1 value)

In this study, the changes in oxyhaemoglobin concentrations in four regions of interest during three motor tasks were collected, and the Beta1 coefficient was calculated using a general linear model (GLM), which reflects the level of cortical activation caused by tasks, to quantify the correlation between brain regions and tasks.

#### 2.3.3 Functional connectivity (FC) analysis of brain regions

Functional connectivity (FC) refers to the temporal correlation between spatially distant neurophysiological events. Brain imaging technology can be used to detect functional communication between multiple regions of the brain (Zheng et al., 2021). In this study, four regions of interest (ROIs) were selected, namely, the left prefrontal cortex (LDLPFC), the right prefrontal cortex (LDLPFC), the RDLPFC, the left supplementary motor area (LSMA) and the right supplementary motor area (RSMA). In the network module of NirSpark software, the changes in the HbO and HbR concentrations measured by the subjects at each time point in the task state were extracted, and the Pearson correlation coefficient between the ROIs was estimated by the correlation method in the time domain. Then, Fisher Z conversion was carried out, and the conversion value was defined as the FC intensity between channels. The FC data were processed with NirSpark software and corrected by the error detection rate (FDR).

#### 2.3.4 Calculation of the centre of gravity value and integral value of each brain region

NirSpark software was used to calculate the centroid value (CV) and integral value (IV) of the ROIs (Takizawa et al., 2014). The integral value represents the changes in cerebral haemodynamics with time during the 30-s task and represents the total size of the haemodynamic signals during the cognitive task, and the unit is millimolar-millimetre (mmol·mm). The larger the integral value is, the greater the content of oxy-Hb during the task. The centre of gravity represents the time of the activation response, which is defined as the time when the positive signal change area under the curve reaches half of the total area of the whole cycle, and the unit is seconds (s). The smaller the centre of gravity value is, the more relaxed and rapid the cortical response of the subject is at the later stage of the task.

### 2.4 Statistical methods

Statistical analysis was carried out using IBM SPSS Statistics 25.0. Descriptive statistics were generated for sex, age, MMSE rating scale score and other data of the included population. The Kolmogorov–Smirnov test was used to analyse whether the parameters conformed to a normal distribution. The measurement data are expressed as the mean and standard deviation (S), and the counting data are expressed as the ratio. The chi-square test was used for comparisons of sex and age, and the independent sample t test was used for comparisons of measurement data between two groups with a normal distribution and homogeneity of variance. One-way analysis of variance (ANOVA) was used to compare the measurement data between tasks in the group, and the Student–Newman–Keuls (SNK) method was used to compare the measurement data between tasks. When the measurement data did not conform to the homogeneity of normal distribution and variance, the rank sum test (Kruskal–Wallis H H) method was adopted. A hypothesis test with *p* < 0.05 was considered statistically significant.

## 3 Results

### 3.1 General information

Ultimately, a total of 45 subjects were included in this study, including 15 in the ELD group and 30 in the YOU group. The results of the general data comparison between the two groups are shown in Table 1, in which the age of the ELD group was significantly greater than that of the YOU group (*p* < 0.001). The MMSE score of the ELD group was significantly lower than that of the YOU group (*p* = 0.009) (see Table 1).

**Table 1:** Distribution of fNIRS channels in the brain.

Channels	Brodmann areas (BA)	Regions of interest (ROI)	Abbreviation
1,2	46	Right Dorsolateral Prefrontal Cortex	RDLPFC
5,6	46	Left Dorsolateral Prefrontal Cortex	LDLPFC
13,14,15,16	6	Right Supplementary Motor Cortex	RSMA
21,22,23,24	6	Left Supplementary Motor Cortex	LSMA

fNIRS, functional near-infrared spectroscopy; ROIs, regions of interest; BA, Brodmann Areas; RDLPFC, right dorsolateral prefrontal cortex; LDLPFC, left dorsolateral prefrontal cortex; RSMA, right supplementary motor cortex; LSMA, left supplementary motor cortex.

### 3.2 Comparison of the performance of the ELD and YOU groups in different tasks

The results of the ELD and YOU groups in different tasks are shown in Table 2. The number of nails inserted in the single motor task in the ELD group was significantly lower than that in the YOU group, and the difference was statistically significant. The numbers of correct and wrong tasks in the ELD group were significantly lower than those in the YOU group, and the difference was statistically significant. The number of nails inserted in the motor–cognitive dual task in the ELD group was significantly lower than that in the YOU group, and the numbers of correct and wrong nails in the ELD group were significantly lower than those in the YOU group. The cost of dual tasks in the ELD group was significantly greater than that in the YOU group, but the difference was not statistically significant (*p* = 0.515) (see Table 2).

**Table 2:** Comparison of the general data of the two groups of subjects.

Subject	ELD	YOU	t	*p*
Sex (male/female)	1/14	11/19	NA	NA
Age	59.73 ± 8.28	22.92 ± 2.79	20.882	<0.001*
Handedness (left/right)	0/15	0/30	NA	NA
MMSE	27.86 ± 3.24	29.63 ± 1.10	−2.720	0.009*

### 3.3 Comparison of brain activation (Beta1 values) between the ELD and YOU groups under different tasks (comparison between groups)

The comparison results of the Beta1 values between the ELD and YOU groups under different tasks are shown in Table 3. The LSMA Beta1 values in the ELD group were significantly lower than those in the YOU group in simple motor tasks. The values of RDLPFC and LDLPFC Beta1 in the ELD group were significantly lower than those in the YOU group. The values of RSMA and LSMA Beta1 in the ELD group were significantly lower than those in the YOU group, and the difference were statistically significant (see Table 3).

**Table 3:** Comparison of nail number, correct and error numbers between young people and old people in the single movement task, single cognitive task and the CMDT.

	ELD	YOU	Statistical values	*p*
Nail number in the single movement task	11.80 ± 1.62	14.24 ± 2.03	*t* = −4.047	<0.001*
Correct number in the single cognitive task	6.24 ± 1.64	10.41 ± 2.76	*t* = −5.362	<0.001*
Error number in the Single task	1.16 ± 1.08	0.44 ± 0.69	*t* = 2.690	0.010*
Double-task nail number	9.20 ± 2.02	11.37 ± 2.91	*t* = −2.586	0.013*
Correct number in the double cognitive task	5.78 ± 1.32	9.68 ± 3.09	*t* = −4.660	<0.001*
Error number in the double cognitive task	1.31 ± 1.13	0.37 ± 0.57	*t* = 3.734	<0.001*
Single motor cost	0.22 ± 0.14	0.20 ± 0.18	*t* = 0.344	0.733
Single cognitive cost	0.05 ± 0.21	0.08 ± 0.21	*t* = −0.502	0.618
Dual-task cost	0.27 ± 0,24	0.29 ± 0.32	*t* = −0.159	0.875

*indicates *p* < 0.05, suggesting a significant difference.

### 3.4 Comparison of brain activation (Beta1 values) between the ELD and YOU groups under different tasks (intragroup comparisons)

As shown in Table 4, the comparative results of the Beta1 values of the subjects in the ELD and YOU groups under different tasks revealed that there was no significant difference in the Beta1 values between the ELD and YOU groups under different tasks (see Table 4).

**Table 4:** Comparison of the activation values (Beta1 values) of the single movement task, single cognitive task and the CMDT between young people and old people.

ROI	Task	Beta1 value		*t*	*p*
		ELD	YOU		
RDLPFC	Task 1	−0.03 ± 0.29	0.04 ± 0.07	−1.254	0.217
	Task 2	−0.01 ± 0.11	0.05 ± 0.07	−2.24	0.031*
	Task 3	−0.00 ± 0.16	0.03 ± 0.06	−0.96	0.343
LDLPFC	Task 1	−0.12 ± 0.43	0.05 ± 0.09	−1.72	0.054
	Task 2	−0.09 ± 0.24	0.04 ± 0.08	−2.65	0.011*
	Task 3	−0.00 ± 0.09	0.03 ± 0.08	−1.45	0.156
RSMA	Task 1	−0.01 ± 0.10	0.05 ± 0.08	−2.43	0.177
	Task 2	0.01 ± 0.18	0.06 ± 0.06	−1.37	0.275
	Task 3	−0.06 ± 0.12	0.04 ± 0.08	−3.41	0.001*
LSMA	Task 1	−0.03 ± 0.22	0.08 ± 0.08	−2.53	0.025*
	Task 2	−0.04 ± 0.30	0.07 ± 0.06	−2.02	0.065
	Task 3	−0.02 ± 0.09	0.08 ± 0.07	−4.00	<0.001*

*indicates *p* < 0.05, suggesting a significant difference. Task 1: single motor task; Task 2: single cognitive task; Task 3: motor–cognitive dual task.

### 3.5 Comparison of brain functional connections between the ELD and YOU groups under different tasks (comparison between groups)

As shown in Table 5, the results of the comparison of the two groups of homologous brain networks revealed that the FC values of the LDLPFC, RSMA and LSMA in the ELD group were significantly lower than those in the YOU group, and the difference was statistically significant. The FC value of the LDLPFC in the ELD group was significantly lower than that in the YOU group, and the difference was statistically significant. The FC values of the LDLPFC and RSMA in the ELD group were significantly lower than those in the YOU group, and the differences were statistically significant (*p* < 0.05).

**Table 5:** Comparison of brain activation between young and old people in the single movement task, the single cognitive task and the CMDT.

ROI	Group	Beta1 value			*F* value	Task 1 vs. Task 3 *p-*value	Task 2 vs. Task 3 *p-*value
		Task 1	Task 2	Task 3			
RDLPFC	ELD	−0.03 ± 0.29	−0.01 ± 0.11	−0.00 ± 0.16	0.057	0.958	0.993
	YOU	0.04 ± 0.07	0.05 ± 0.07	0.03 ± 0.06	0.980	0.852	0.431
LDLPFC	ELD	−0.12 ± 0.43	−0.09 ± 0.24	−0.00 ± 0.09	0.549	0.621	0.597
	YOU	0.05 ± 0.09	0.04 ± 0.08	0.03 ± 0.08	0.347	0.820	0.906
RSMA	ELD	−0.01 ± 0.10	0.01 ± 0.18	−0.06 ± 0.12	1.271	0.228	0.429
	YOU	0.05 ± 0.08	0.06 ± 0.06	0.04 ± 0.08	0.670	0.966	0.566
LSMA	ELD	−0.03 ± 0.22	−0.04 ± 0.30	−0.02 ± 0.09	0.024	0.992	0.974
	YOU	0.08 ± 0.08	0.07 ± 0.07	0.08 ± 0.07	0.133	0.988	0.926

*indicates *p* < 0.05, suggesting a significant difference. Task 1: single motor task; Task 2: single cognitive task; Task 3: motor–cognitive dual task.

**Figure 3:**
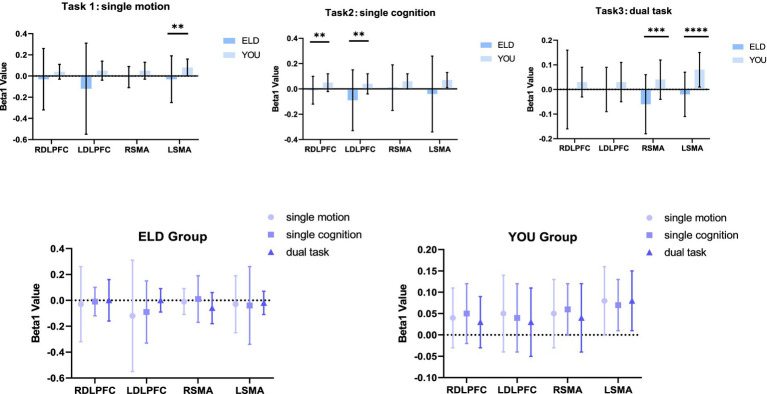
Brain activation (Beta1 values) between the ELD and YOU groups under different tasks. There was no significant difference in the Beta1 values between the ELD and YOU groups under different tasks.

**Figure 4:**
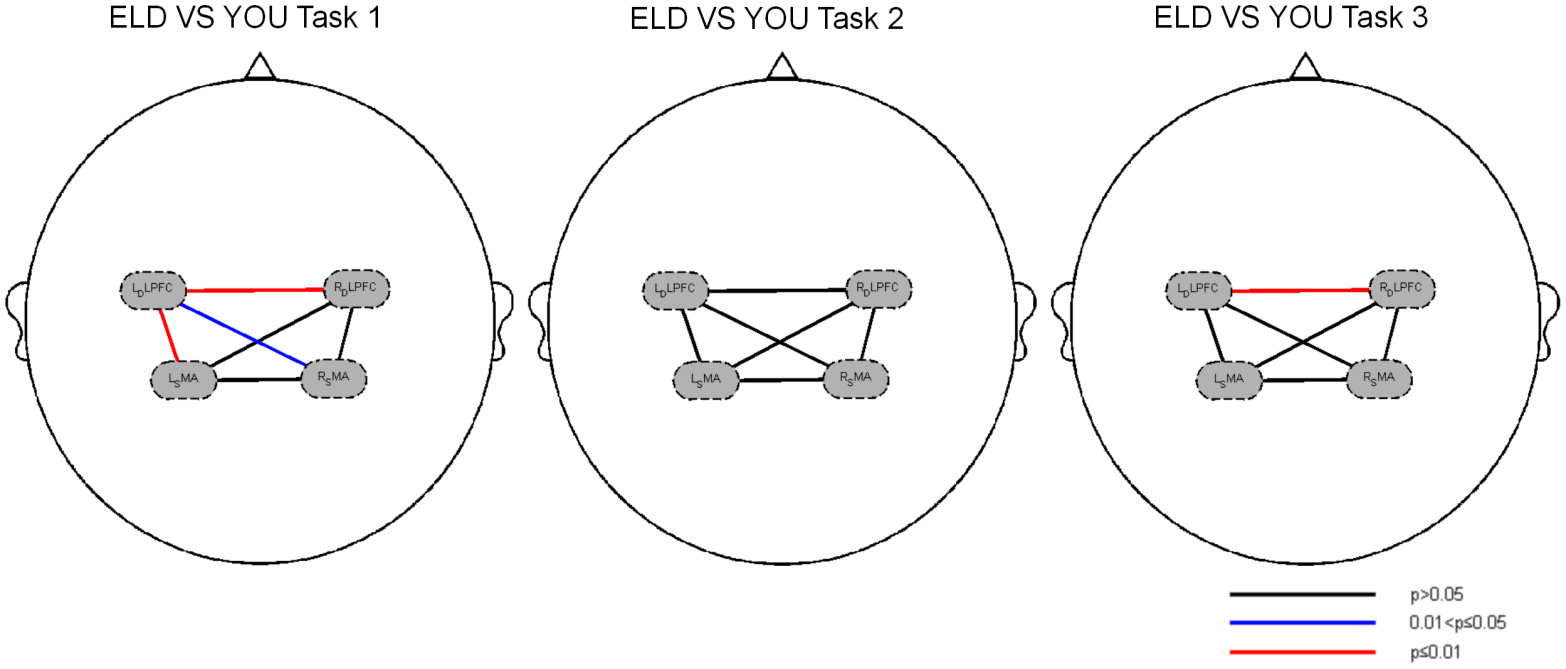
Brain functional connections between the ELD and YOU groups under different tasks. Task 1: single motor task, task 2: single cognitive task, task 3: motor-cognitive dual task. In the single motor task, the functional connectivity of RDLPFC–LDLPFC, LDLPFC–LSMA and LDLPFC–RSMA in the ELD group were significantly lower than those in the YOU group. In the motor-cognitive dual task, the functional connectivity of RDLPFC-LDLPFC in the ELD group was also lower, but there was no significant difference between the two groups.

In the comparison of the two groups of heterogeneous brain networks, the FC values of RDLPFC–LDLPFC, LDLPFC–LSMA and LDLPFC–RSMA in the ELD group were significantly lower than those in the YOU group, and the differences were statistically significant. The FC value of the RDLPFC–LDLPFC in the ELD group was significantly lower than that in the YOU group, and the difference was statistically significant. In the single cognitive task, there was no significant difference in the FC values of the ROIs from different sources between the ELD group and the YOU group (see Table 5; Figures 3, 4).

### 3.6 Comparison of brain function connections between the ELD and YOU groups under different tasks (intragroup comparisons)

As shown in Table 6, the comparison of homologous brain networks in the ELD group revealed that the FC value of the RSMA (*p* = 0.041) was greater than that of the motor–cognitive dual task, and the difference was statistically significant. The comparison of homologous brain networks in the YOU group revealed that the FC value of the LSMA (*p* = 0.011) was significantly greater than that of the motor–cognitive dual task, and the FC value of the LSMA (*p* = 0.021) was significantly greater than that of the motor–cognitive dual task in the single cognitive task.

**Table 6:** Brain functional connections between young people and elderly people in the single movement task, the single cognitive task and the CMDT (comparison between groups).

Homogenous ROIs	Task	FC value		t	*p*
		ELD	YOU		
RDLPFC	Task 1	0.30 ± 0.45	0.54 ± 0.25	−1.940	0.068
	Task 2	0.45 ± 0.29	0.47 ± 0.35	−0.188	0.851
	Task 3	0.26 ± 0.31	0.415 ± 0.34	−1.437	0.158
LDLPFC	Task 1	0.23 ± 0.36	0.53 ± 0.29	−2.972	0.005*
	Task 2	0.35 ± 0.26	0.57 ± 0.28	−2.527	0.015*
	Task 3	0.23 ± 0.35	0.45 ± 0.31	−2.156	0.037*
RSMA	Task 1	0.39 ± 0.20	0.58 ± 0.22	−2.821	0.007*
	Task 2	0.54 ± 0.21	0.56 ± 0.24	−0.130	0.897
	Task 3	0.35 ± 0.20	0.52 ± 0.28	−2.037	0.048*
LSMA	Task 1	0.42 ± 0.24	0.61 ± 0.20	−2.801	0.008*
	Task 2	0.53 ± 0.25	0.59 ± 0.22	−0.919	0.363
	Task 3	0.37 ± 0.23	0.43 ± 0.26	−0.820	0.417

Heterologous ROIs	Task	FC value		*t*	*p*
		ELD	YOU		
RDLPFC-LDLPFC	Task 1	0.21 ± 0.21	0.44 ± 0.22	−3.499	0.001*
	Task 2	0.41 ± 0.16	0.48 ± 0.23	−1.130	0.265
	Task 3	0.19 ± 0.22	0.40 ± 0.24	−2.779	0.008*
RDLPFC-LSMA	Task 1	0.22 ± 0.23	0.35 ± 0.24	−1.757	0.086
	Task 2	0.39 ± 0.25	0.39 ± 0.23	−0.053	0.958
	Task 3	0.21 ± 0.19	0.29 ± 0.25	−0.972	0.336
RDLPFC-RSMA	Task 1	0.27 ± 0.31	0.39 ± 0.20	−1.614	0.114
	Task 2	0.44 ± 0.23	0.39 ± 0.23	0.608	0.546
	Task 3	0.25 ± 0.24	0.27 ± 0.18	−0.220	0.827
LDLPFC-LSMA	Task 1	0.15 ± 0.21	0.38 ± 0.22	−3.407	0.001*
	Task 2	0.30 ± 0.22	0.37 ± 0.24	−0.968	0.339
	Task 3	0.22 ± 0.18	0.28 ± 0.26	−0.752	0.456
LDLPFC-RSMA	Task 1	0.18 ± 0.22	0.37 ± 0.26	−2.449	0.018*
	Task 2	0.33 ± 0.21	0.37 ± 0.25	−0.544	0.589
	Task 3	0.20 ± 0.16	0.25 ± 0.22	−0.655	0.516
LSMA-RSMA	Task 1	0.34 ± 0.18	0.47 ± 0.25	−1.783	0.082
	Task 2	0.45 ± 0.23	0.53 ± 0.21	−1.062	0.294
	Task 3	0.27 ± 0.17	0.39 ± 0.25	−1.743	0.088

*indicates *p* < 0.05, suggesting a significant difference. Task 1: single motor task; Task 2: single cognitive task; Task 3: motor–cognitive dual task.

**Figure 5:**
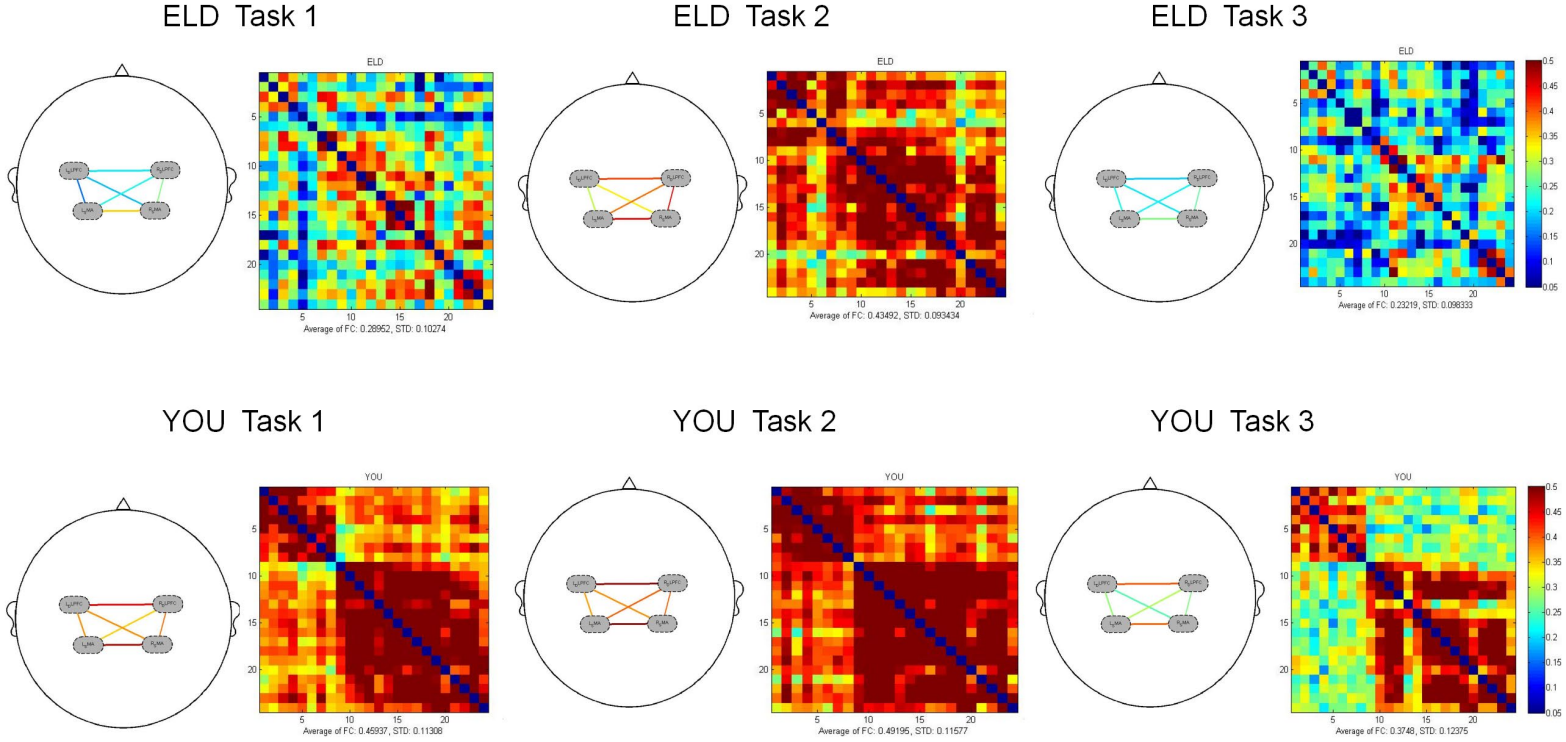
Brain activation between the ELD and YOU groups under different tasks. Task 1: single motor task, task 2: single cognitive task, task 3: motor-cognitive dual task. The brain activation of 3 tasks in the ELD group were all lower than those in the YOU group. The value of the motor-cognitive dual task in the ELD group was significantly much lower.

**Figure 6:**
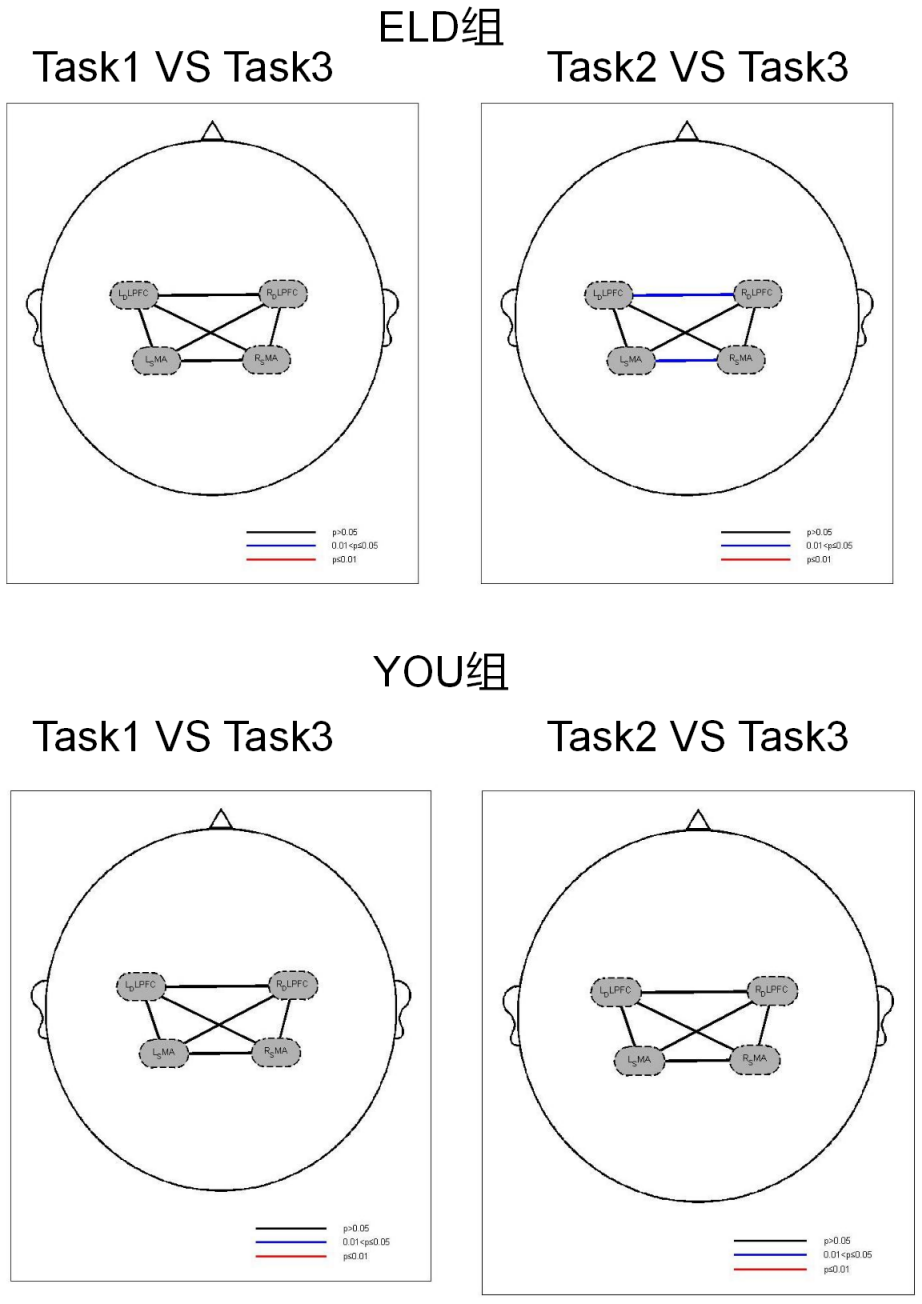
Brain function connections between young and elderly people in different tasks. Task 1: single motor task, Task 2: single cognitive task, Task 3: motor-cognitive dual task. There were no significant difference between single tasks and dual task. Both groups showed the same results.

A comparison of heterogeneous brain networks in the ELD group revealed that the FC values of the RDLPFC–LDLPFC (*p* = 0.014) and LSMA–RSMA (*p* = 0.038) were significantly greater than those of the motor–cognitive tasks. There was no significant difference in the FC values between the motor, cognitive and motor–cognitive dual tasks in the comparison of heterogeneous brain networks in the YOU group (see Table 6 and Figures 5, 6).

### 3.7 Comparison of brain centroid value between the ELD and YOU groups under different tasks (comparison between groups)

The comparison results of the centre of gravity values of the ROIs between the ELD and YOU groups in the different tasks are shown in Table 7. There was no significant difference in all the centre of gravity values of the ROIs in the single cognitive and single motor tasks. In the motor–cognitive dual task, the centre of gravity in the LDLPFC of the ELD group was significantly lower than that of the YOU group, and the difference was statistically significant (*p* < 0.05) (see Table 7).

**Table 7:** Brain function connections between young and elderly people in the single movement task, the single cognitive task and the CMDT (intragroup comparisons).

Homologous	Group	Task 1	Task 2	Task 3	*F* value	Task 1 vs. Task 3 *p*-value	Task 2 vs. Task 3 *p*-value
RDLPFC	ELD	0.30 ± 0.45	0.45 ± 0.29	0.26 ± 0.31	1.285	0.954	0.296
	YOU	0.54 ± 0.25	0.47 ± 0.35	0.41 ± 0.34	1.285	0.250	0.696
LDLPFC	ELD	0.23 ± 0.36	0.35 ± 0.26	0.23 ± 0.35	0.724	0.999	0.545
	YOU	0.53 ± 0.29	0.57 ± 0.28	0.45 ± 0.31	1.310	0.534	0.257
RSMA	ELD	0.39 ± 0.20	0.54 ± 0.21	0.35 ± 0.20	3.573	0.885	0.041*
	YOU	0.58 ± 0.22	0.55 ± 0.24	0.52 ± 0.28	0.507	0.576	0.847
LSMA	ELD	0.42 ± 0.24	0.53 ± 0.25	0.37 ± 0.23	1.683	0.846	0.185
	YOU	0.61 ± 0.20	0.59 ± 0.22	0.43 ± 0.26	5.437	0.011*	0.021*

Heterologous	Group	Task 1	Task 2	Task 3	*F* value	Task 1 vs. Task 3 *p*-value	Task 2 vs. Task 3 *p*-value
RDLPFC-LDLPFC	ELD	0.21 ± 0.21	0.41 ± 0.16	0.19 ± 0.22	5.441	0.984	0.014*
	YOU	0.44 ± 0.22	0.48 ± 0.23	0.40 ± 0.24	0.917	0.767	0.370
RDLPFC-LSMA	ELD	0.22 ± 0.23	0.39 ± 0.25	0.21 ± 0.19	2.926	1.000	0.102
	YOU	0.35 ± 0.24	0.39 ± 0.23	0.29 ± 0.25	1.465	0.572	0.210
RDLPFC-RSMA	ELD	0.27 ± 0.31	0.44 ± 0.23	0.25 ± 0.24	2.304	0.992	0.147
	YOU	0.39 ± 0.20	0.39 ± 0.23	0.27 ± 0.18	3.627	0.061	0.052
LDLPFC-LSMA	ELD	0.15 ± 0.21	0.30 ± 0.22	0.22 ± 0.18	2.019	0.586	0.570
	YOU	0.38 ± 0.22	0.37 ± 0.24	0.28 ± 0.26	1.558	0.252	0.320
LDLPFC-RSMA	ELD	0.18 ± 0.22	0.33 ± 0.21	0.20 ± 0.16	2.407	0.940	0.214
	YOU	0.37 ± 0.26	0.37 ± 0.25	0.25 ± 0.22	2.639	0.114	0.128
LSMA-RSMA	ELD	0.34 ± 0.18	0.45 ± 0.23	0.27 ± 0.17	3.297	0.563	0.038*
	YOU	0.47 ± 0.25	0.53 ± 0.21	0.39 ± 0.25	2.316	0.404	0.088

*indicates *p* < 0.05, suggesting a significant difference. Task 1: single motor task; Task 2: single cognitive task; Task 3: motor–cognitive dual task.

### 3.8 Comparison of brain centroid value between the ELD and YOU groups under different tasks (intragroup comparisons)

The comparison of centroid value in the ELD group revealed that there was no significant difference in the centroid value between the ROIs in the single motor task and the motor–cognitive dual task. In the single cognitive task, the gravity centre value of the RSMA (*p* = 0.041) was lower than that in the motor–cognitive dual task, and the difference was statistically significant. The comparison of barycentre values in the YOU group revealed that the barycentre value of the RDLPFC (*p* < 0.001) in the single motor task was significantly greater than that in the motor–cognitive dual task, and the barycentre value of the RDLPFC (*p* = 0.024) in the single cognitive task was significantly greater than that in the motor–cognitive dual task (see Table 8).

**Table 8:** Comparison of brain centroid value between young and old people in the single movement task, the single cognitive task and the CMDT (comparison between groups).

ROI	Task	Centroid value		*t*	*p*
		ELD	YOU		
RDLPFC	Task 1	27.70 ± 12.57	21.98 ± 9.51	1.707	0.095
	Task 2	26.09 ± 6.03	28.08 ± 8.00	−0.850	0.400
	Task 3	26.33 ± 11.24	31.69 ± 8.99	−1.733	0.090
LDLPFC	Task 1	29.00 ± 12.59	25.33 ± 7.91	1.069	0.291
	Task 2	24.71 ± 9.33	27.12 ± 9.77	−0.791	0.433
	Task 3	22.88 ± 13.67	30.79 ± 8.85	−2.345	0.024*
RSMA	Task 1	28.46 ± 10.52	24.20 ± 7.91	1.521	0.136
	Task 2	24.00 ± 7.10	24.46 ± 7.09	−0.222	0.926
	Task 3	30.66 ± 12.51	26.03 ± 9.24	1.406	0.160
LSMA	Task 1	27.92 ± 6.85	25.87 ± 5.86	1.043	0.303
	Task 2	27.80 ± 10.96	24.77 ± 5.64	1.234	0.244
	Task 3	28.01 ± 6.92	24.33 ± 6.41	1.720	0.085

*indicates *p* < 0.05, suggesting a significant difference. Task 1: single motor task; Task 2: single cognitive task; Task 3: motor–cognitive dual task.

### 3.9 Comparison of brain integral values between the ELD and YOU groups under different tasks (comparison between groups)

The comparison results of the scores of the subjects in the ELD and YOU groups under different tasks are shown in Table 9. There was no significant difference in the scores of the ELD and YOU groups under different tasks among the ROIs (see Table 9).

**Table 9:** Comparison of brain centroid value between young and old people in the single movement task, the single cognitive task and the CMDT (intragroup comparisons).

ROI	Group	Centroid value			*F* value	Task 1 vs. Task 3 *p-*value	Task 2 vs. Task 3 *p-*value
		Task 1	Task 2	Task 3			
RDLPFC	ELD	28.47 ± 11.40	26.82 ± 5.54	27.19 ± 9.92	1.285	0.954	0.296
	YOU	21.98 ± 9.51	28.08 ± 8.00	31.69 ± 8.99	9.222	<0.001*	0.024*
LDLPFC	ELD	30.73 ± 13.27	24.90 ± 9.07	25.06 ± 12.77	0.724	0.999	0.545
	YOU	25.33 ± 7.91	27.12 ± 9.77	30.79 ± 8.85	2.564	0.073	0.299
RSMA	ELD	29.18 ± 10.43	24.20 ± 6.23	30.49 ± 11.33	3.573	0.885	0.041*
	YOU	24.20 ± 7.91	24.46 ± 7.09	26.03 ± 9.24	0.444	0.660	0.736
LSMA	ELD	28.14 ± 7.60	26.85 ± 10.57	27.73 ± 6.94	1.683	0.846	0.185
	YOU	25.87 ± 5.86	24.77 ± 5.64	24.33 ± 6.41	0.528	0.581	0.958

*indicates *p* < 0.05, suggesting a significant difference. Task 1: single motor task; Task 2: single cognitive task; Task 3: motor–cognitive dual task.

### 3.10 Comparison of brain integral values between the ELD and YOU groups under different tasks (comparison between groups)

**Figure 7:**
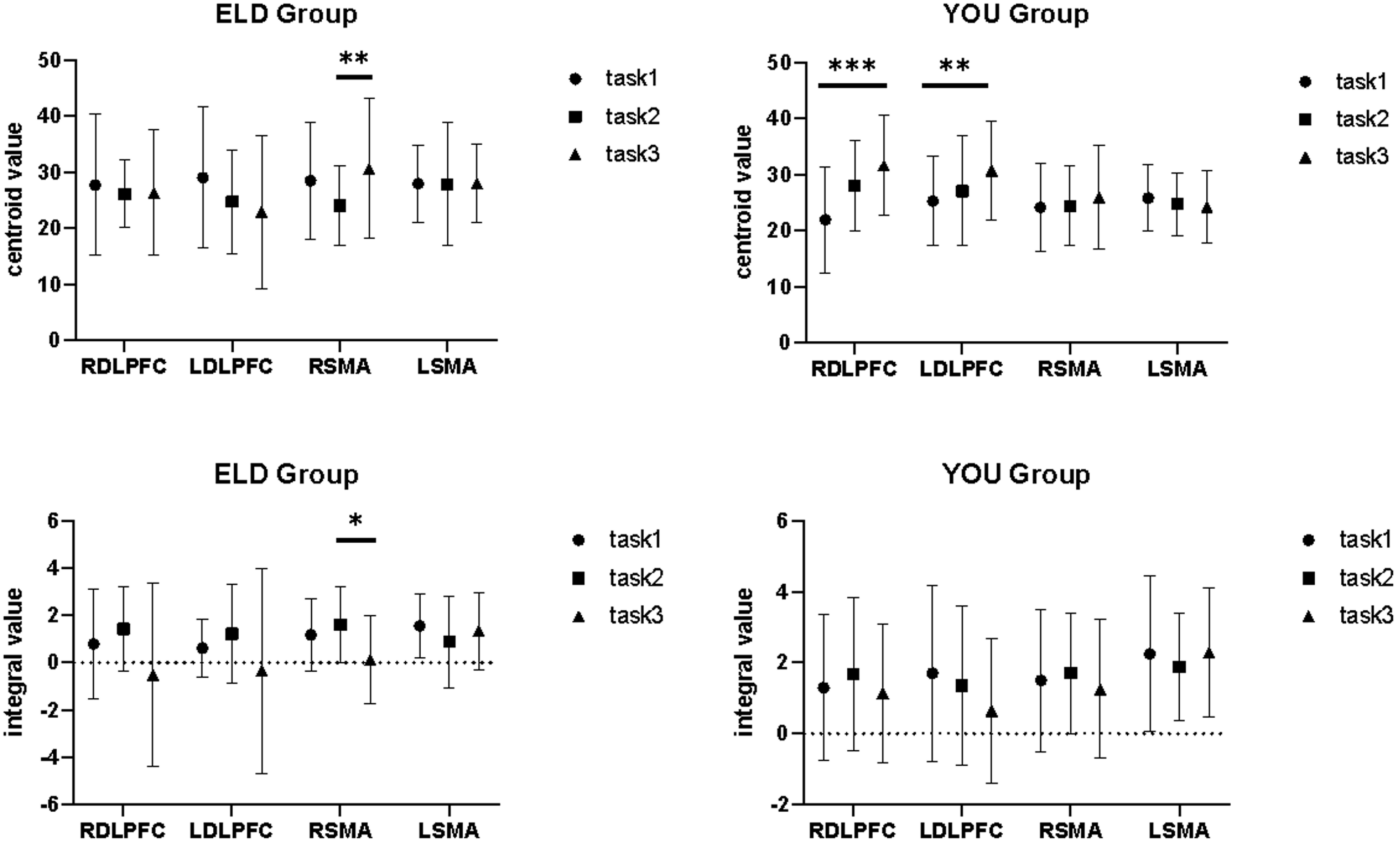
Brain integral values and centroid values between the ELD and YOU groups under different tasks. Task 1: single motor task, Task 2: single cognitive task, Task 3: motor-cognitive dual task. In the ELD group, only the centroid and integral values of the RSMA were found to be significantly different between the single and dual cognitive tasks. In the YOU group, only the centroid and integral values of the DLPFC were found to be significantly different between single and dual tasks.

**Figure 8:**
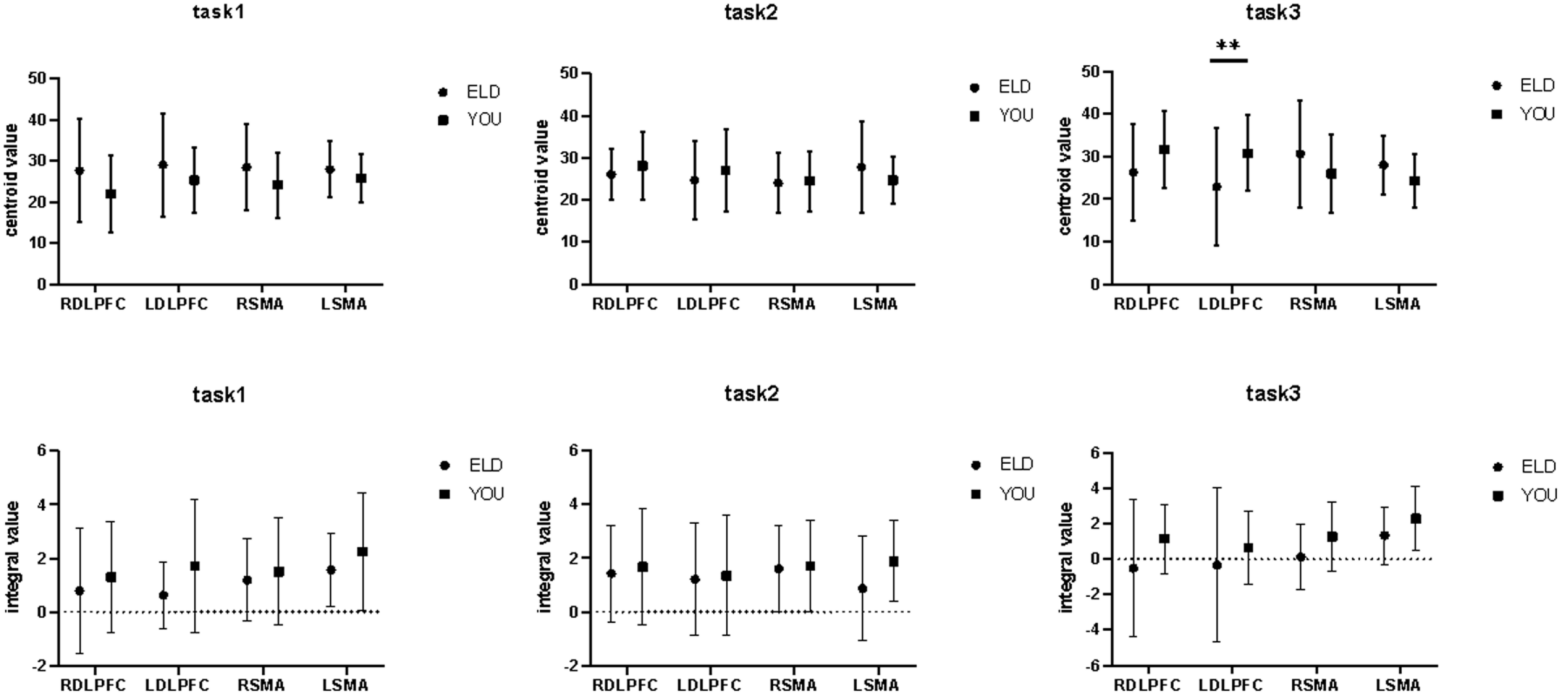
Brain integral and centroid values between the ELD and YOU groups under different tasks. Task 1: single motor task, Task 2: single cognitive task, Task 3: motor-cognitive dual task. Comparisons between the two groups revealed significant differences in the centroid values of the LDLPFC under dual tasks.

As shown in Table 10, the comparison results of internal product values in the ELD group revealed that there was no significant difference in the integral values of each ROI in the single motor task compared with those in the motor–cognitive dual task, and the integral value of RSMA (*p* = 0.050) in the single cognitive task was significantly lower than that in the motor–cognitive dual task. The comparison results of internal product scores in the YOU group revealed that there was no significant difference between the single motor task and the single cognitive task (see Table 10; Figures 7, 8).

**Table 10:** Comparison of brain integral values between young and old people in the single movement task, the single cognitive task and the CMDT (comparison between groups).

ROI	Task	Integral value		*t*	*p*
		ELD	YOU		
RDLPFC	Task 1	0.79 ± 2.32	1.30 ± 2.06	−0.763	0.450
	Task 2	1.43 ± 1.79	1.68 ± 2.15	−0.390	0.690
	Task 3	−0.52 ± 3.87	1.14 ± 1.95	−1.924	0.061
LDLPFC	Task 1	0.62 ± 1.24	1.70 ± 2.49	−1.587	0.120
	Task 2	1.22 ± 2.09	1.35 ± 2.25	−0.188	0.852
	Task 3	−0.34 ± 4.34	0.64 ± 2.05	−1.037	0.305
RSMA	Task 1	1.19 ± 1.54	1.50 ± 2.00	−0.514	0.610
	Task 2	1.61 ± 1.60	1.70 ± 1.70	−0.170	0.866
	Task 3	0.12 ± 1.86	1.26 ± 1.96	−1.866	0.069
LSMA	Task 1	1.56 ± 1.36	2.25 ± 2.19	−1.103	0.276
	Task 2	0.88 ± 1.93	1.89 ± 1.51	−1.940	0.059
	Task 3	1.34 ± 1.63	2.30 ± 1.82	−1.727	0.083

*indicates *p* < 0.05, suggesting a significant difference. Task 1: single motor task; Task 2: single cognitive task; Task 3: motor–cognitive dual task.

### 3.11 Correlation analysis between RDLPFC centroid value of dual task and dual-task costs in ELD and YOU groups

**Figure 9:**
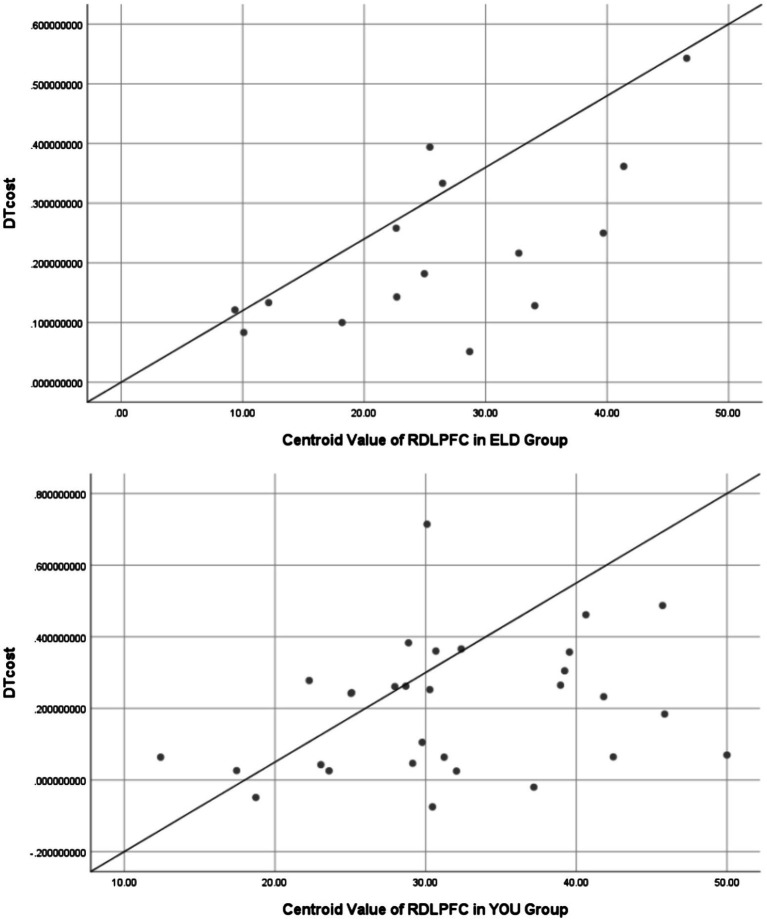
Scatter plots between RDLPFC centroid value of dual task and dual-task costs in ELD and YOU groups. There was a positive linear correlation between RDLPFC centroid value and dual-task cost in both groups.

As shown in Table 11, the correlation coefficient between the dual task RDLPFC centroid value and dual task cost was statistically significant in the ELD group (r = 0.009, *p* < 0.05), but no significant difference was found in the YOU group (see Figure 9; Table 12).

**Table 11:** Comparison of brain integral values between young and old people in the single movement task, the single cognitive task and the CMDT (comparison between groups).

ROI	Group	Integral value			*F* value	Task 1 vs. Task 3 *p-*value	Task 2 vs. Task 3 *p-*value
		Task 1	Task 2	Task 3			
RDLPFC	ELD	0.79 ± 2.32	1.43 ± 1.79	−0.52 ± 3.87	1.888	0.416	0.149
	YOU	1.30 ± 2.06	1.68 ± 2.15	1.14 ± 1.95	0.549	0.951	0.566
LDLPFC	ELD	0.62 ± 1.24	1.22 ± 2.09	−0.34 ± 4.34	1.131	0.634	0.305
	YOU	1.70 ± 2.49	1.35 ± 2.25	0.64 ± 2.05	1.705	0.170	0.447
RSMA	ELD	1.19 ± 1.54	1.61 ± 1.60	0.12 ± 1.86	3.147	0.199	0.050*
	YOU	1.50 ± 2.00	1.70 ± 1.70	1.26 ± 1.96	0.403	0.881	0.644
LSMA	ELD	1.56 ± 1.36	0.88 ± 1.93	1.34 ± 1.63	0.672	0.924	0.730
	YOU	2.25 ± 2.19	1.89 ± 1.51	2.30 ± 1.82	0.421	0.994	0.679

*indicates *p* < 0.05, suggesting a significant difference. Task 1: single motor task; Task 2: single cognitive task; Task 3: motor–cognitive dual task.

**Table 12:** Correlation analysis of dual task RDLPFC centroid value and dual task cost between ELD group and YOU group.

Subject	ELD group	YOU group
Centroid value of RDLPFC	26.33 ± 11.24	31.69 ± 8.99
Dual task cost	0.21 ± 0.14	0.20 ± 0.19
*r*	0.651	0.268
*p*	0.009*	0.153

*indicates *p* < 0.05, suggesting a significant difference.

## 4 Discussion

### 4.1 Influence of brain ageing on motor–cognitive task performance

In this study, people aged 50–80 years were defined as the elderly population (ELD group) based on the following considerations. Although WTO defines people over the age of 65 as elderly, researches have showed that brain structure and function begin to show significant age-related changes from age 50, including reduced gray matter volume, decreased white matter integrity, and reduced metabolic rate (Stark and Pakkenberg, 2004). This may be associated with early cognitive and motor decline. Second, individuals over 50 years of age have shown a decline in executive function and reduced efficiency in allocating neural resources in behavioral tasks, which is closely related to the core goal of this study—to explore age-related changes in functional connectivity in the brain. Therefore, we extended the age range of seniors to over 50 years old.

In this study, SMA (Brodmann 6 area) were selected as ROIs, and their functions related to motor planning and execution were mainly focused. However, there are limitations to this option. Brodmann 6 area includes pre-supplementary motor area (pre-SMA) and supplementary motor area (SMA proper), which have different functions: pre-SMA is involved in complex movement sequences and cognitive control (such as task switching), while SMA proper is more involved in motor preparation and execution. Treating SMA as a single whole may result in the loss of function-specific information, such as the inability to distinguish the effects of cognitive load on pre-SMA. Besides, Brodmann 6 area has functional connectivity with premotor cortex (PMC) and primary motor cortex (M1), which jointly regulates fine movement.

As expected, there was a significant difference in the MMSE baseline scores between the elderly and young groups. Additionally, the elderly group performed significantly worse on tasks such as the number of nails and the continuous subtraction of 7 s compared to the young group. Notably, the MMSE scores of the elderly participants remained within the normal range, indicating that although their cognitive function appeared normal on the MMSE, their cognitive performance had declined in terms of behavioral tasks. Compared to the young group, the elderly group exhibited a decline in fine hand movements and working memory, likely due to slower brain processing speeds. The aging brain is reflected in both scale scores and functional performance, with differences between single and dual tasks linked to deficits in executive function and reductions in both cognitive and motor abilities (Collyer et al., 2022; Montero-Odasso et al., 2020; Wang et al., 2025).

Interestingly, while there were no significant differences in the dual-task (DT) cost between the elderly and young groups, the DT cost was significantly higher in the elderly group. This suggests that when cognitive load is increased, it can impair motor performance more markedly in the elderly, particularly in those experiencing cognitive decline, further elucidating the cognitive deterioration linked to brain aging. Therefore, combining motor-cognitive dual-task results with MMSE cognitive scale assessments could offer a more comprehensive understanding of brain aging. However, there is a lack of objective evidence examining the mechanisms of brain aging from a behavioral perspective. To address this gap, this study incorporates synchronous fNIRS technology to explore the functional interactions between motor-and cognitive-related brain regions.

### 4.2 Effects of brain ageing on brain activation and functional connectivity

Currently, there are two main hypotheses that explain age-related changes in brain activity: the dedifferentiation hypothesis and the compensation hypothesis. The dedifferentiation hypothesis posits that brain regions involved in task execution lose functional specificity with age (Park et al., 2004; Rajah and D'esposito, 2005). Some researchers argue that this pattern begins with a decline in dopaminergic regulation, leading to increased neural noise and less distinct cortical representations. In contrast, the compensation hypothesis suggests that elderly individuals may recruit higher levels of activity in certain brain regions to compensate for functional deficits elsewhere in the brain. Our findings align more closely with the dedifferentiation hypothesis.

Fine motor tasks are crucial for evaluating motor function. The Purdue Nail Board task, widely used in rehabilitation, assesses manual dexterity and fine motor skills (Bakhshipour et al., 2019). Our results show that the elderly group performed worse than the young control group, with a decrease in supplementary motor area (SMA) activation on the contralateral side. Previous studies have indicated that the SMA plays a key role in motor planning and is involved in movements generated and controlled by the individual, rather than external stimuli (Li et al., 2024). For instance, Chen et al. (2024) reported widespread bilateral sensorimotor cortex activation in stroke patients during Purdue Nail Board tasks, with higher-functioning patients showing more extensive bilateral activation during both one-and two-handed motor tasks. Similarly, Horenstein et al. (2009) showed that bilateral networks are crucial for planning and executing motor tasks in healthy individuals. These findings suggest that fine motor tasks require greater neural recruitment, and activation of the contralateral hemisphere may serve a compensatory role in improving motor function.

However, our study revealed significantly lower activation of the left SMA in the elderly, which may suggest that age-related declines in fine motor ability are linked to reduced activation in specific motor areas. The SMA, located in the supplementary motor area, is crucial for the difficulty and accuracy of motor task execution. In the elderly, poor task performance may reflect the loss of functional specificity in the left SMA, resulting in decreased brain region activation during tasks.

Cognitive control, which involves adjusting behavior to meet internal goals in a dynamic environment (Luna et al., 2015), includes processes like maintaining or updating goal-relevant information (working memory) and suppressing irrelevant information. The continuous subtraction task, which involves arithmetic processing, is closely linked to cognitive control processes (Hinault and Lemaire, 2016a; Hinault and Lemaire, 2016b). In our study, elderly individuals showed significantly lower activation of bilateral dorsolateral prefrontal cortex (DLPFC) areas compared to younger participants during single cognitive tasks. This suggests that the brain function of healthy older adults is generally reduced compared to younger individuals. Similar findings have been reported in other studies, such as Nagel et al. (2011), who observed that increased prefrontal cortex activity related to load is accompanied by decreased functional coupling between the PFC and premotor cortex. Madden et al. (2010) also reported that although brain activity levels between young and old adults were similar, the functional connectivity in the elderly was lower.

However, when performing motor-cognitive dual tasks, our findings show that while bilateral SMA activation decreased in the elderly compared to the young group, activation in cognitive brain regions (including the DLPFC) did not decrease. This suggests that older adults may engage compensatory mechanisms, recruiting greater activity in certain brain regions to compensate for functional deficits. Typically, this compensatory activation is observed in the frontal lobe. The prefrontal cortex plays a pivotal role in allocating brain resources effectively, coordinating subgoals to achieve the ultimate goal. In this study, the decrease in activation of bilateral DLPFCs in elderly participants could reflect cognitive resource limitations resulting from brain aging, as well as the heterogeneity of cognitive decline. The observed decline in cognitive function in older adults is likely contributing to decreased DLPFC activation.

Further comparison of brain functional connectivity across three task conditions revealed declines in connectivity in the elderly compared to younger adults. This finding is consistent with prior studies using fMRI to assess brain network connectivity in healthy aging individuals (Li et al., 2023). Our analysis showed that functional connectivity between the left DLPFC and other brain regions was reduced, particularly during single motor tasks. The DLPFC is part of the executive network, while the SMA belongs to the motor network. Compared to the default mode network of normal adults, these networks are typically less active at rest (Yeo et al., 2011). In some cases, decreased activity in task-related areas is positively correlated with brain atrophy in those areas (Brassen et al., 2009; Rajah et al., 2011). Longitudinal studies show that grey matter volume in the frontal and temporal lobes decreases linearly throughout adulthood (from 20 to 80 years of age) (Raz et al., 2007). Areas like the DLPFC and orbital prefrontal cortex are particularly sensitive to age-related changes (Raz et al., 1997), and these areas correlate with declines in cognitive functions such as working memory, situational memory, and the ability to manage distractions. Therefore, excessive inhibition of functional connectivity in these regions in elderly subjects is more likely to be a result of brain atrophy. Furthermore, the reduced functional connectivity in the elderly may be linked to age-related declines in white matter integrity, which is crucial for transmitting information between brain regions (Serbruyns et al., 2015; Sullivan et al., 2010). Previous studies have shown that individuals with high white matter burden exhibit decreased DLPFC activity and impaired brain connectivity. Reduced DLPFC connectivity has also been identified as a predictor of cognitive decline in elderly individuals. For example, Toepper et al. (2014) observed that elderly individuals with greater functional connectivity in the right DLPFC outperformed those with lower connectivity in spatial working memory tasks. Similarly, Steffener et al. (2012) found that poor performance in oral working memory tasks was associated with changes in functional connectivity between brain networks.

### 4.3 Influence of brain ageing on the center of gravity and integral value of brain regions

The integral value reflects changes in cerebral hemodynamics over time during a 30-s task and indicates the total size of the hemodynamic signals during the task. Larger integral values correspond to greater activation of brain regions. In comparing the two groups, no significant differences were found in the integral values of any brain regions between the elderly and young groups. This may be due to the relative simplicity of the Purdue Nail Board and minus-7 tasks for healthy individuals, meaning the activation thresholds of the involved brain regions were not high, leading to no obvious differences between groups.

The barycenter value represents the activation reaction time, defined as the point where the positive signal change area under the HbO concentration curve reaches half of the total area of the entire cycle. In general, smaller barycenter values indicate faster changes in cerebral blood flow at the end of the task, suggesting quicker and more efficient responses in those regions. Our results showed that although both groups exhibited similar activation levels, the elderly participants had reduced barycentric values in their regions of interest compared to the young group. A significant difference was observed in the barycentric values of the left DLPFC during dual tasks. We believe that the faster decrease in cerebral blood flow in the elderly leads to a slower recovery of brain activation, thereby shifting the barycenter forward in the HbO curve.

We propose that the left DLPFC, which plays a central role in brain connectivity, is particularly sensitive to age-related declines in cerebral blood flow. This brain region’s sensitivity to age-related changes may explain why it exhibited significant differences in blood flow decrease and recovery following task completion.

### 4.4 New contributions to the mechanisms of brain aging

Our study found that the activation of the LSMA in the single motor task was significantly lower in the elderly than in the young, and the activation of the DLPFC was also significantly weakened in the single cognitive task. In addition, the elderly not only showed reduced activation of bilateral auxiliary motor areas (RSMA and LSMA) during dual tasks, but their functional connections (such as LDLPFC-RSMA and LSMA-RSMA) were also significantly weaker than those of younger adults. This seems to fit with the dedifferentiation hypothesis, that is, the functional specificity of brain regions decreases with age (Rieck et al., 2020). In addition, a previous narrative review study showed that the integrity of the brain’s frontal regions, specifically the prefrontal cortex, is critical for maintaining cognitive and functional abilities in later life (Udina et al., 2019). Our study showed that the activation of DLPFC in the elderly was decreased to varying degrees when they completed single or dual task. This may suggest that decreased DLPFC activation is one of the key features of brain aging.

Furthermore, we investigated whether changes in dual task cost were correlated with changes in cerebral hemodynamics. The change of barycentric value can indirectly reflect the cerebral vascular regulation ability. Our study found that the center of gravity value in the elderly was significantly reduced in the dual task, indicating a slower dynamic response rate of cerebral blood flow, which may be related to age-related decline in cerebral vascular regulation. The dynamic response of blood flow may be slowed down due to decreased vascular elasticity or weakened neurovascular coupling. Correlation analysis showed that the centroid value of RDLPFC in dual task was positively correlated with the cost of dual task, especially the correlation coefficient between the two in the ELD group was statistically significant, which means that the longer the RDLPFC reaction time to reach maximum oxygen saturation, the greater decrease in the ability to perform the dual task. We suggest that the RDLPFC centroid value can be used as a sensitive marker of hemodynamic changes during aging. It also extends the application of fNIRS in the assessment of aging-related cerebral hemodynamic changes.

Although our main results come from healthy people, it is important to note that these findings may apply to patient groups, especially those with mild cognitive impairment or conscious cognitive decline. As an exploratory analysis, we found that as the difficulty and number of tasks increased during healthy aging, the degree of brain activation and functional connectivity decreased, and the task cost increased. As one of the key features of aging, the degree of DLPFC activation can help clinicians intervene early in the process of aging and prevent cognitive decline during aging. Early intervention can prevent or reduce the occurrence of mild cognitive impairment or conscious cognitive decline, and delay the aging process of such people. Future studies need to include different patient groups (such as mild cognitive impairment and conscious cognitive decline) and larger sample sizes, in order to further determine the role and mechanism of DLPFC in cognitive impairment and its potential clinical application.

## 5 Study limitations

There are several potential limitations in this study that need to explain.

Frist, there are limitation to this definition of age and gender. The large age span of 50–80 years may lead to higher heterogeneity within the group. In addition, some 50-year-old individuals may not yet show significant aging characteristics, and their functional decline may be related to other confounding factors (such as lifestyle, comorbidities), which may affect the interpretation of the results. The gender distribution of the elderly sample in this study is uneven, which may affect the generalization of the results. Future studies need to include gender-balanced older cohorts to verify the generalizations of the current findings.

Second, the small sample size may account for the lack of significant differences between the two groups in terms of dual-task costs, Beta1 values between ROIs during different tasks, and barycentric values across single cognitive and motor tasks. These limitations reduce the generalizability of the findings. Future studies should further expand the sample size for analysis.

Besides, the study did not cover PMC or M1, which may ignore the dynamic changes of motor network synergies, especially the resource allocation in dual tasks. In the future, high-resolution imaging (such as fMRI) can be used to subdivide Brodmann 6 area and extend ROI to PMC, M1 and other areas, so as to more comprehensively analyse the aging mechanism of motor-cognitive network.

The PPT task, which focused on right-handed tasks, did not include left-handed, two-handed, or assembly subdomain tasks, limiting the comprehensiveness of motor task evaluation and the resulting brain activation and connectivity measures. Future studies should investigate the activation and functional connectivity of relevant brain regions during left-hand and two-hand cooperative fine motor tasks.

## 6 Conclusion

Conclusion Overall, this study reveals the negative effects of brain aging on cognitive and motor function, and demonstrates changes in functional dedifferentiation and compensatory mechanisms of the aging brain. Although older individuals are able to compensate for declining function by increasing brain region activation in certain tasks, weakened brain functional connectivity and changes in brain blood flow remain important markers of the aging process. Therefore, the combination of MMSE, motor—cognitive dual task assessment and brain functional imaging can reveal the mechanism of brain aging more comprehensively. Future research is needed to further explore the specific mechanisms of these changes and consider larger sample study designs in order to provide stronger evidence for early intervention in cognitive and motor disorders in older adults, which provides a new theoretical basis for how to intervene or delay the aging process through exercise and cognitive tasks in clinical practice.

## Data Availability

The raw data supporting the conclusions of this article will be made available by the authors, without undue reservation.
