# Supplementary material for: Functional connectivity and characteristics of cortical brain networks of elderly individuals under different motor cognitive tasks based on functional near-infrared spectroscopy
Source: Front Hum Neurosci. 2025 Apr 24;19:1563338. doi: 10.3389/fnhum.2025.1563338 (PMC12058795; doi:10.3389/fnhum.2025.1563338)
Supplement: Supplementary file 1 [file Table_1.DOCX]

**Supplementary Material：**

**（1）Results of the subject effect analysis of each task feature value and group and gender**

Table 1 Analysis of Between-Subjects Effects for Group and Gender on Single Cognitive Task Centroid Value

|  | ROI | Type III Sum of Squares | df | Mean Square | *F* | *p* |
| --- | --- | --- | --- | --- | --- | --- |
| Group | RDLPFC | 120.870 | 1 | 120.870 | 2.426 | 0.127 |
|  | LDLPFC | 0.106 | 1 | 0.106 | 0.001 | 0.972 |
|  | RSMA | 49.746 | 1 | 49.746 | 1.008 | 0.321 |
|  | LSMA | 0.002 | 1 | 0.002 | ＜0.001 | 0.995 |
| Gender | RDLPFC | 5.192 | 1 | 5.192 | 0.104 | 0.749 |
|  | LDLPFC | 167.109 | 1 | 167.109 | 1.962 | 0.169 |
|  | RSMA | 29.523 | 1 | 29.523 | 0.598 | 0.444 |
|  | LSMA | 24.206 | 1 | 24.206 | 0.390 | 0.536 |
| Group*Gender | RDLPFC | 170.514 | 1 | 170.514 | 3.422 | 0.072 |
|  | LDLPFC | 1.869 | 1 | 1.869 | 0.22 | 0.883 |
|  | RSMA | 69.555 | 1 | 69.555 | 1.409 | 0.242 |
|  | LSMA | 57.052 | 1 | 57.052 | 0.918 | 0.343 |

Table 2 Analysis of Between-Subjects Effects for Group and Gender on Single Cognitive Task Integral Value

|  | ROI | Type III Sum of Squares | df | mean square | *F* | *p* |
| --- | --- | --- | --- | --- | --- | --- |
| Group | RDLPFC | 0.130 | 1 | 0.130 | 0.031 | 0.861 |
|  | LDLPFC | 0.412 | 1 | 0.412 | 0.084 | 0.774 |
|  | RSMA | 0.201 | 1 | 0.201 | 0.069 | 0.794 |
|  | LSMA | 1.368 | 1 | 1.368 | 0.478 | 0.493 |
| Gender | RDLPFC | 3.259 | 1 | 3.259 | 0.772 | 0.385 |
|  | LDLPFC | 0.241 | 1 | 0.241 | 0.049 | 0.826 |
|  | RSMA | 0.210 | 1 | 0.210 | 0.072 | 0.790 |
|  | LSMA | 0.853 | 1 | 0.853 | 0.298 | 0.588 |
| Group*Gender | RDLPFC | 0.100 | 1 | 0.100 | 0.024 | 0.879 |
|  | LDLPFC | 1.364 | 1 | 1.364 | 0.277 | 0.601 |
|  | RSMA | 0.055 | 1 | 0.055 | 0.019 | 0.892 |
|  | LSMA | 0.471 | 1 | 0.471 | 0.165 | 0.687 |

Table 3 Analysis of Between-Subjects Effects for Group and Gender on Single Cognitive Task Activation Value

|  | ROI | Type III Sum of Squares | df | mean square | *F* | *p* |
| --- | --- | --- | --- | --- | --- | --- |
| Group | RDLPFC | 0.006 | 1 | 0.006 | 0.694 | 0.410 |
|  | LDLPFC | 0.019 | 1 | 0.019 | 0.809 | 0.374 |
|  | RSMA | 0.015 | 1 | 0.015 | 1.077 | 0.305 |
|  | LSMA | 0.032 | 1 | 0.032 | 1.030 | 0.316 |
| Gender | RDLPFC | 0.003 | 1 | 0.003 | 0.317 | 0.577 |
|  | LDLPFC | 0.012 | 1 | 0.012 | 0.534 | 0.469 |
|  | RSMA | 0.003 | 1 | 0.003 | 0.217 | 0.644 |
|  | LSMA | 0.001 | 1 | 0.001 | 0.024 | 0.879 |
| Group*Gender | RDLPFC | 0.002 | 1 | 0.002 | 0.198 | 0.659 |
|  | LDLPFC | 0.010 | 1 | 0.010 | 0.439 | 0.511 |
|  | RSMA | ＜0.001 | 1 | ＜0.001 | 0.034 | 0.854 |
|  | LSMA | 0.001 | 1 | 0.001 | 0.019 | 0.892 |

Table 4 Analysis of Between-Subjects Effects for Group and Gender on Single Motor Task Centroid Value

|  | ROI | Type III Sum of Squares | df | mean square | *F* | *p* |
| --- | --- | --- | --- | --- | --- | --- |
| Group | RDLPFC | 6.408 | 1 | 6.408 | 0.057 | 0.813 |
|  | LDLPFC | 14.793 | 1 | 14.793 | 0.121 | 0.730 |
|  | RSMA | 8.815 | 1 | 8.815 | 0.103 | 0.750 |
|  | LSMA | 0.844 | 1 | 0.844 | 0.026 | 0.873 |
| Gender | RDLPFC | 163.243 | 1 | 163.243 | 1.443 | 0.237 |
|  | LDLPFC | 28.520 | 1 | 28.520 | 0.233 | 0.632 |
|  | RSMA | 76.608 | 1 | 76.608 | 0.964 | 0.332 |
|  | LSMA | 135.397 | 1 | 135.397 | 4.126 | 0.049 |
| Group*Gender | RDLPFC | 41.056 | 1 | 41.056 | 0.363 | 0.550 |
|  | LDLPFC | 3.048 | 1 | 3.048 | 0.025 | 0.875 |
|  | RSMA | 11.251 | 1 | 11.251 | 0.142 | 0.709 |
|  | LSMA | 0.556 | 1 | 0.556 | 0.017 | 0.897 |

Table 5 Analysis of Between-Subjects Effects for Group and Gender on Single Motor Task Integral Value

|  | ROI | Type III Sum of Squares | df | mean square | *F* | *p* |
| --- | --- | --- | --- | --- | --- | --- |
| Group | RDLPFC | 0.074 | 1 | 0.074 | 0.013 | 0.909 |
|  | LDLPFC | 3.812 | 1 | 3.812 | 0.569 | 0.455 |
|  | RSMA | 1.913 | 1 | 1.913 | 0.451 | 0.505 |
|  | LSMA | 0.919 | 1 | 0.919 | 0.171 | 0.682 |
| Gender | RDLPFC | 0.776 | 1 | 0.776 | 0.139 | 0.711 |
|  | LDLPFC | 0.239 | 1 | 0.239 | 0.036 | 0.851 |
|  | RSMA | 0.148 | 1 | 0.148 | 0.035 | 0.853 |
|  | LSMA | 0.425 | 1 | 0.425 | 0.079 | 0.780 |
| Group*Gender | RDLPFC | 0.280 | 1 | 0.280 | 0.050 | 0.824 |
|  | LDLPFC | 8.358 | 1 | 8.358 | 1.248 | 0.271 |
|  | RSMA | 2.304 | 1 | 2.304 | 0.544 | 0.465 |
|  | LSMA | 1.704 | 1 | 1.704 | 0.317 | 0.577 |

Table 6 Analysis of Between-Subjects Effects for Group and Gender on Single Motor Task Activation Value

|  | ROI | Type III Sum of Squares | df | mean square | *F* | *p* |
| --- | --- | --- | --- | --- | --- | --- |
| Group | RDLPFC | ＜0.001 | 1 | ＜0.001 | 0.015 | 0.904 |
|  | LDLPFC | 0.023 | 1 | 0.023 | 0.335 | 0.566 |
|  | RSMA | ＜0.001 | 1 | ＜0.001 | 0.002 | 0.969 |
|  | LSMA | 0.001 | 1 | 0.001 | 0.056 | 0.814 |
| Gender | RDLPFC | 0.030 | 1 | 0.030 | 1.011 | 0.321 |
|  | LDLPFC | 0.065 | 1 | 0.065 | 0.944 | 0.337 |
|  | RSMA | 0.020 | 1 | 0.020 | 2.821 | 0.101 |
|  | LSMA | 0.047 | 1 | 0.047 | 2.333 | 0.134 |
| Group*Gender | RDLPFC | 0.006 | 1 | 0.006 | 0.202 | 0.655 |
|  | LDLPFC | 0.011 | 1 | 0.011 | 0.154 | 0.697 |
|  | RSMA | 0.002 | 1 | 0.002 | 0.301 | 0.586 |
|  | LSMA | 0.024 | 1 | 0.024 | 1.191 | 0.282 |

Table 7 Analysis of Between-Subjects Effects for Group and Gender on Dual Task Centroid Value

|  | ROI | Type III Sum of Squares | df | mean square | *F* | *p* |
| --- | --- | --- | --- | --- | --- | --- |
| Group | RDLPFC | 104.041 | 1 | 104.041 | 1.079 | 0.305 |
|  | LDLPFC | 61.190 | 1 | 61.190 | 0.523 | 0.474 |
|  | RSMA | 95.987 | 1 | 95.987 | 0.851 | 0.362 |
|  | LSMA | 0.212 | 1 | 0.212 | 0.005 | 0.945 |
| Gender | RDLPFC | 18.374 | 1 | 18.374 | 0.190 | 0.665 |
|  | LDLPFC | 29.726 | 1 | 29.726 | 0.254 | 0.617 |
|  | RSMA | 0.625 | 1 | 0.625 | 0.006 | 0.941 |
|  | LSMA | 45.087 | 1 | 45.087 | 1.024 | 0.317 |
| Group*Gender | RDLPFC | 16.355 | 1 | 16.355 | 0.170 | 0.683 |
|  | LDLPFC | 83.538 | 1 | 83.538 | 0.714 | 0.403 |
|  | RSMA | 11.004 | 1 | 11.004 | 0.098 | 0.756 |
|  | LSMA | 56.725 | 1 | 56.725 | 1.289 | 0.263 |

Table 8 Analysis of Between-Subjects Effects for Group and Gender on Dual Task Integral Value

|  | ROI | Type III Sum of Squares | df | mean square | *F* | *p* |
| --- | --- | --- | --- | --- | --- | --- |
| Group | RDLPFC | 0.142 | 1 | 0.142 | 0.019 | 0.891 |
|  | LDLPFC | 0.051 | 1 | 0.051 | 0.006 | 0.941 |
|  | RSMA | 2.630 | 1 | 2.630 | 0.677 | 0.415 |
|  | LSMA | 0.003 | 1 | 0.003 | 0.001 | 0.974 |
| Gender | RDLPFC | 4.819 | 1 | 4.819 | 0.646 | 0.426 |
|  | LDLPFC | 4.118 | 1 | 4.118 | 0.445 | 0.508 |
|  | RSMA | 0.328 | 1 | 0.328 | 0.084 | 0.773 |
|  | LSMA | 2.482 | 1 | 2.482 | 0.801 | 0.376 |
| Group*Gender | RDLPFC | 13.609 | 1 | 13.609 | 1.823 | 0.184 |
|  | LDLPFC | 6.460 | 1 | 6.460 | 0.699 | 0.408 |
|  | RSMA | 0.226 | 1 | 0.226 | 0.058 | 0.811 |
|  | LSMA | 6.054 | 1 | 6.054 | 1.954 | 0.170 |

Table 9 Analysis of Between-Subjects Effects for Group and Gender on Dual Task Activation Value

|  | ROI | Type III Sum of Squares | *df* | mean square | *F* | *p* |
| --- | --- | --- | --- | --- | --- | --- |
| Group | RDLPFC | 0.001 | 1 | 0.001 | 0.060 | 0.808 |
|  | LDLPFC | 0.002 | 1 | 0.002 | 0.233 | 0.639 |
|  | RSMA | 0.017 | 1 | 0.017 | 2.026 | 0.162 |
|  | LSMA | 0.007 | 1 | 0.007 | 1.047 | 0.312 |
| Gender | RDLPFC | 0.001 | 1 | 0.001 | 0.048 | 0.827 |
|  | LDLPFC | 0.004 | 1 | 0.004 | 0.543 | 0.465 |
|  | RSMA | 0.011 | 1 | 0.011 | 1.264 | 0.267 |
|  | LSMA | 0.019 | 1 | 0.019 | 3.301 | 0.089 |
| Group*Gender | RDLPFC | 0.002 | 1 | 0.002 | 0.184 | 0.670 |
|  | LDLPFC | ＜0.001 | 1 | ＜0.001 | 0.025 | 0.874 |
|  | RSMA | 0.001 | 1 | 0.001 | 0.068 | 0.796 |
|  | LSMA | 0.012 | 1 | 0.012 | 1.793 | 0.188 |

**（2）Results of coherence analysis in different brain regions**

| Homologous | Group | Task 1 | Task 2 | Task 3 | F  Value | Task 1 vs. Task 3  *p* Value | Task 2 vs. Task 3  *p* Value |
| --- | --- | --- | --- | --- | --- | --- | --- |
| RDLPFC | ELD | 0.63±0.34 | 0.74±0.28 | 0.59±0.32 | 0.881 | 0.627 | 0.931 |
|  | YOU | 0.76±0.28 | 0.75±0.29 | 0.71±0.33 | 0.338 | 0.978 | 0.710 |
| LDLPFC | ELD | 0.53±0.34 | 0.71±0.36 | 0.56±0.30 | 1.264 | 0.310 | 0.966 |
|  | YOU | 0.69±0.32 | 0.72±0.32 | 0.70±0.33 | 0.085 | 0.920 | 0.997 |
| RSMA | ELD | 0.71±0.21 | 0.72±0.21 | 0.53±0.21 | 4.304 | 0.980 | 0.049* |
|  | YOU | 0.72±0.21 | 0.73±0.22 | 0.74±0.22 | 0.092 | 0.946 | 0.912 |
| LSMA | ELD | 0.68±0.20 | 0.76±0.20 | 0.68±0.20 | 0.751 | 0.544 | 0.999 |
|  | YOU | 0.70±0.22 | 0.71±0.21 | 0.75±0.22 | 0.336 | 0.989 | 0.721 |
| Heterologous |  | | | | | | |
| RDLPFC-LDLPFC | ELD | 0.69±0.24 | 0.73±0.34 | 0.57±0.30 | 1.151 | 0.326 | 0.014* |
|  | YOU | 0.78±0.23 | 0.80±0.21 | 0.57±0.30 | 0.389 | 0.767 | 0.370 |
| RDLPFC-LSMA | ELD | 0.60±0.37 | 0.71±0.25 | 0.57±0.26 | 0.918 | 0.407 | 0.102 |
|  | YOU | 0.64±0.32 | 0.70±0.31 | 0.77±0.24 | 1.492 | 0.231 | 0.210 |
| RDLPFC-RSMA | ELD | 0.73±0.28 | 0.67±0.31 | 0.52±0.33 | 1.839 | 0.172 | 0.147 |
|  | YOU | 0.59±0.35 | 0.66±0.36 | 0.72±0.29 | 1.076 | 0.345 | 0.052 |
| LDLPFC-LSMA | ELD | 0.51±0.40 | 0.64±0.32 | 0.46±0.32 | 0.965 | 0.389 | 0.570 |
|  | YOU | 0.67±0.34 | 0.69±0.33 | 0.71±0.34 | 0.115 | 0.892 | 0.320 |
| LDLPFC-RSMA | ELD | 0.52±0.37 | 0.57±0.36 | 0.55±0.32 | 0.091 | 0.913 | 0.214 |
|  | YOU | 0.56±0.30 | 0.63±0.34 | 0.67±0.28 | 1.059 | 0.351 | 0.128 |
| LSMA-RSMA | ELD | 0.70±0.30 | 0.75±0.28 | 0.60±0.36 | 0.945 | 0.397 | 0.038* |
|  | YOU | 0.64±0.33 | 0.82±0.28 | 0.76±0.29 | 2.809 | 0.066 | 0.088 |

| Homogenous ROIs | Task | FC value | | t | *p* |
| --- | --- | --- | --- | --- | --- |
|  |  | ELD | YOU |  |  |
| RDLPFC | Task 1 | 0.63±0.34 | 0.76±0.28 | -1.404 | 0.168 |
|  | Task 2 | 0.74±0.28 | 0.75±0.29 | -0.166 | 0.908 |
|  | Task 3 | 0.59±0.32 | 0.71±0.33 | -1.102 | 0.277 |
| LDLPFC | Task 1 | 0.53±0.34 | 0.69±0.32 | -1.527 | 0.134 |
|  | Task 2 | 0.71±0.36 | 0.72±0.32 | -0.092 | 0.927 |
|  | Task 3 | 0.56±0.30 | 0.70±0.33 | -1.330 | 0.191 |
| RSMA | Task 1 | 0.71±0.21 | 0.72±0.21 | -0.063 | 0.950 |
|  | Task 2 | 0.72±0.21 | 0.73±0.22 | -0.109 | 0.914 |
|  | Task 3 | 0.53±0.21 | 0.74±0.22 | -3.052 | 0.004* |
| LSMA | Task 1 | 0.68±0.20 | 0.70±0.22 | -0.382 | 0.704 |
|  | Task 2 | 0.76±0.20 | 0.71±0.21 | 0.660 | 0.513 |
|  | Task 3 | 0.68±0.20 | 0.75±0.22 | -1.022 | 0.312 |
| Heterologous ROIs |  | | | | |
| RDLPFC-LDLPFC | Task 1 | 0.69±0.24 | 0.78±0.23 | -1.182 | 0.244 |
|  | Task 2 | 0.73±0.34 | 0.80±0.21 | -0.893 | 0.377 |
|  | Task 3 | 0.57±0.30 | 0.83±0.24 | -3.128 | 0.003* |
| RDLPFC-LSMA | Task 1 | 0.60±0.37 | 0.64±0.32 | -0.406 | 0.687 |
|  | Task 2 | 0.71±0.25 | 0.70±0.31 | 0.152 | 0.880 |
|  | Task 3 | 0.57±0.26 | 0.77±0.24 | -2.619 | 0.012* |
| RDLPFC-RSMA | Task 1 | 0.73±0.28 | 0.59±0.35 | 1.282 | 0.2.7 |
|  | Task 2 | 0.67±0.31 | 0.66±0.36 | 0.083 | 0.934 |
|  | Task 3 | 0.52±0.33 | 0.72±0.29 | -2.066 | 0.045* |
| LDLPFC-LSMA | Task 1 | 0.51±0.40 | 0.67±0.34 | -1.437 | 0.158 |
|  | Task 2 | 0.64±0.32 | 0.69±0.33 | -0.486 | 0.629 |
|  | Task 3 | 0.46±0.32 | 0.71±0.34 | -2.733 | 0.009* |
| LDLPFC-RSMA | Task 1 | 0.52±0.37 | 0.56±0.30 | -0.413 | 0.682 |
|  | Task 2 | 0.57±0.36 | 0.63±0.34 | -0.565 | 0.575 |
|  | Task 3 | 0.55±0.32 | 0.67±0.28 | -1.304 | 0.199 |
| LSMA-RSMA | Task 1 | 0.70±0.30 | 0.64±0.33 | 0.606 | 0.548 |
|  | Task 2 | 0.75±0.28 | 0.82±0.28 | -0.697 | 0.490 |
|  | Task 3 | 0.60±0.36 | 0.76±0.29 | -1.616 | 0.113 |
